# Specialized Peptidoglycan Hydrolases Sculpt the Intra-bacterial Niche of Predatory *Bdellovibrio* and Increase Population Fitness

**DOI:** 10.1371/journal.ppat.1002524

**Published:** 2012-02-09

**Authors:** Thomas R. Lerner, Andrew L. Lovering, Nhat Khai Bui, Kaoru Uchida, Shin-Ichi Aizawa, Waldemar Vollmer, R. Elizabeth Sockett

**Affiliations:** 1 Centre for Genetics and Genomics, School of Biology, University of Nottingham, Medical School, Nottingham, United Kingdom; 2 School of Biosciences, University of Birmingham, Birmingham, United Kingdom; 3 The Centre for Bacterial Cell Biology, Medical School, Newcastle University, Newcastle upon Tyne, United Kingdom; 4 Department of Life Sciences, Prefectural University of Hiroshima, Shobara, Hiroshima, Japan; Harvard University, United States of America

## Abstract

*Bdellovibrio* are predatory bacteria that have evolved to invade virtually all Gram-negative bacteria, including many prominent pathogens. Upon invasion, prey bacteria become rounded up into an osmotically stable niche for the *Bdellovibrio*, preventing further superinfection and allowing *Bdellovibrio* to replicate inside without competition, killing the prey bacterium and degrading its contents. Historically, prey rounding was hypothesized to be associated with peptidoglycan (PG) metabolism; we found two *Bdellovibrio* genes, *bd0816* and *bd3459*, expressed at prey entry and encoding proteins with limited homologies to conventional *dacB*/PBP4 DD-endo/carboxypeptidases (responsible for peptidoglycan maintenance during growth and division). We tested possible links between Bd0816/3459 activity and predation. Bd3459, but not an active site serine mutant protein, bound β-lactam, exhibited DD-endo/carboxypeptidase activity against purified peptidoglycan and, importantly, rounded up *E. coli* cells upon periplasmic expression. A ΔBd0816 ΔBd3459 double mutant invaded prey more slowly than the wild type (with negligible prey cell rounding) and double invasions of single prey by more than one *Bdellovibrio* became more frequent. We solved the crystal structure of Bd3459 to 1.45 Å and this revealed predation-associated domain differences to conventional PBP4 housekeeping enzymes (loss of the regulatory domain III, alteration of domain II and a more exposed active site). The Bd3459 active site (and by similarity the Bd0816 active site) can thus accommodate and remodel the various bacterial PGs that *Bdellovibrio* may encounter across its diverse prey range, compared to the more closed active site that “regular” PBP4s have for self cell wall maintenance. Therefore, during evolution, *Bdellovibrio* peptidoglycan endopeptidases have adapted into secreted predation-specific proteins, preventing wasteful double invasion, and allowing activity upon the diverse prey peptidoglycan structures to sculpt the prey cell into a stable intracellular niche for replication.

## Author Summary

*Bdellovibrio bacteriovorus* is a small predatory bacterium that invades other bacteria including pathogens of humans and animals. *Bdellovibrio* digest the pathogens from within, growing at their expense. *Bdellovibrio* do not attack human, plant or animal cells and so could be applied as “living antibiotics”. Here we have discovered how *Bdellovibrio* evolved to live inside other bacteria. Evolution has changed (normally housekeeping) genes called *dacB*s so that their products recognise and modify the different cell walls of a wide range of bacteria. Their action sculpts the cell walls of the invaded bacteria to make a stable “home” for the *Bdellovibrio*, inside which it kills them. We know the structure and activity of the enzymes and that mutants without them are not as efficient predators. This is relevant to antibacterial therapies because the predatory DacB enzymes themselves act against bacteria and are also a key factor in *Bdellovibrio* cells being live predators of pathogens.

## Introduction

*Bdellovibrio bacteriovorus* are small predatory bacteria which invade the periplasm of other Gram-negative bacteria, round up these (typically formerly rod-shaped) prey bacteria into spherical, osmotically stable structures called bdelloplasts, kill the prey and replicate within them. Constructing the bdelloplast gives *Bdellovibrio* a “private” source of food in a niche which does not leak nutrients to competitors outside. Essential to bdelloplast “sculpting” is modification, by the *Bdellovibrio*, of the prey cell wall peptidoglycan in the prey bacterium's periplasm. We set out to determine the *Bdellovibrio* enzymes responsible for this.

During normal growth of bacteria, penicillin-binding-proteins (PBPs) synthesize and remodel the essential peptidoglycan layer of their own cell envelope [1], [2]. In Gram-negative proteobacteria, the net-like peptidoglycan is formed of repeating units of *N*-acetylglucosamine and *N*-acetylmuramic acid residues which are cross-linked via short peptides containing D-amino acids [3]. The peptidoglycan gives osmotic stability and is required to maintain bacterial cell shape. Class A PBPs are peptidoglycan synthases polymerizing peptidoglycan monomers (lipid II) into nascent chains and forming peptide cross-links, while class B PBPs have only peptide cross-linking activity and are often specific for cell elongation or division [1], [4]. For the cell wall to grow as the bacteria elongate and divide, there is a need to remodel the peptidoglycan to incorporate more cell wall structural units. Thus bacteria have various peptidoglycan hydrolases which cleave their own peptidoglycan [5] and it is proposed that PBPs may act within multi-enzyme complexes in this process [6].

Class C PBPs (or low molecular mass PBPs) are implicated in this “cutting” and remodelling of peptidoglycan and they include DD-carboxypeptidases, which remove the terminal D-Ala residue from pentapeptides, and DD-endopeptidases which hydrolyze the D-Ala-*meso*-Dap cross-links [1], [7]. Gram-negative bacteria have many PBPs [1], *Escherichia coli* K-12 has 12 for example [8], they all bind substrate analogues like penicillin; and all members share a common evolutionary origin. The crystal structure determination of PBPs in recent years [9]–[11] has illuminated some of their functional details [4], [6]. All PBPs have a conserved active site serine residue in a Ser-X-X-Lys motif, along with conserved Ser-X-Asn and Lys-Thr-Gly motifs [1]. Several PBPs can have the same peptidoglycan hydrolase activity in a single bacterium; this redundancy explains why the deletion of single PBP genes usually does not give a strong phenotype in lab conditions [12], yet the remodelling of the cell wall is a vital process for bacterial growth and division [5].

In this study we have discovered a novel use of some evolutionarily diversified class C PBPs, by the predatory bacterium *Bdellovibrio bacteriovorus* HD100 when they invade prey and construct the osmotically stable bdelloplast from the prey cell. In 1978, Thomashow and Rittenberg carried out a thoughtful biochemical analysis of peptidoglycan enzyme activities which they could detect as mixtures during prey-bacterial invasion by *Bdellovibrio*. They hypothesised that lytic transglycosylases, which degrade the glycan chains in peptidoglycan, would be involved in prey entry and that cell wall peptidases would be involved in the rounding of the invaded prey cell into the osmotically stable bdelloplast [13].

We followed up Thomashow and Rittenberg's hypotheses [13]. In a transcriptomic study we noted the induction of expression of two highly homologous genes, *bd0816* and *bd3459*, in *Bdellovibrio* 30 minutes into the prey invasion process [14]. These genes showed homology to *dacB* genes encoding PBP4 DD-endo/carboxy-peptidases, peptidoglycan-remodelling enzymes of proteobacteria. Targeted mutagenesis has shown us that their encoded products round up the prey into a bdelloplast and concomitantly prevents a wasteful tailgating invasion of one prey cell by two *Bdellovibrio*. The crystal structure of Bd3459 shows that it and Bd0816 are modified PBP4 versions lacking the domain III that is common to housekeeping PBP4 proteins that act only on a bacterium's own peptidoglycan. We propose that several structural modifications from the PBP4 archetype confer predatory function, acting on a wide range of prey-bacterial PGs, to the Bd3459 and Bd0816 enzymes.

## Results

### *B. bacteriovorus* HD100 has three *dacB*-like genes, two of which are expressed during the invasion stage of the predatory cycle

**Figure 1:**
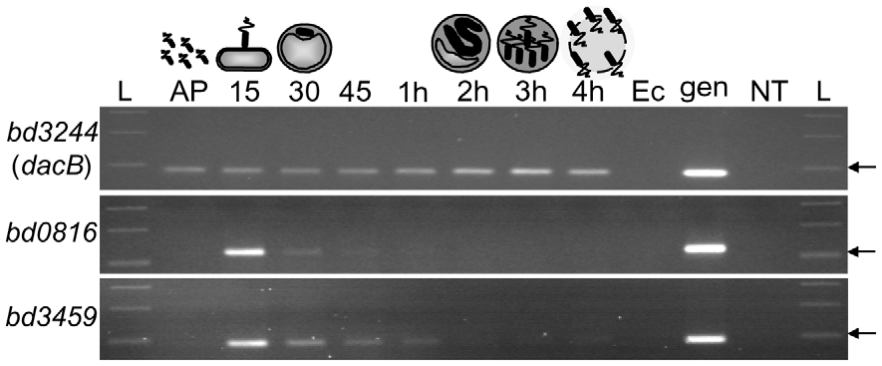
Reverse-transcriptase PCR showing relative transcriptional expression of the three *B. bacteriovorus* HD100 *dacB* homologues. RNA was isolated at the indicated time points during one round of synchronous *Bdellovibrio* infection of *E. coli* host cells, and primers specific to the genes *bd3244* (*dacB*), *bd0816* and *bd3459* (*dacB*-like) were used to amplify an approximately 100 bp internal fragment from each transcript if present. *bd3244* expression shows constitutive expression across the *Bdellovibrio* lifecycle, (including 2–3 hour septation period) as would be expected for a “peptidoglycan-housekeeping” gene. *bd0816* and *bd3459* show a strong peak of expression at 15 minutes post-infection, when *Bdellovibrio* has attached to prey cells and is beginning to invade. The expression diminishes rapidly at the 45 minute to 1 hour time point once invasion is complete and the prey bdelloplast is formed, and before septation. This is indicative of predation-specific expression. AP = Attack phase (free-swimming *Bdellovibrio*); 15, 30, 45 = minutes since start of infection (attachment to and rounding of *E. coli* host cell into a bdelloplast structure); 1 h, 2 h, 3 h, 4 h = hours since start of infection (host cell resources being degraded and used for *Bdellovibrio* growth into a filament, followed by septation into multiple progeny and eventually lysis from host cell); Ec = *E. coli* S17-1 RNA (no *Bdellovibrio* control); gen = *B. bacteriovorus* HD100 genomic DNA (positive control); NT = No template control; L = NEB 100 bp DNA ladder, arrowed is 100 bp.

Transcriptomics [14] followed up by reverse transcriptase PCR (RT-PCR) of *Bdellovibrio* gene expression in total RNA sampled across the predatory cycle showed that *dacB*-like genes *bd0816* and *bd3459*, but not a third *dacB* homologue *bd3244*, were specifically upregulated at the 15–30 minute time points (Figure 1). Gene *bd3244*, which encodes a predicted protein with the highest sequence identity to *E. coli* “housekeeping” PBP4, showed constitutive expression throughout the predatory cycle, as would be expected for a predicted class C PBP that would be involved in the housekeeping function of constant peptidoglycan turnover in the *Bdellovibrio* itself. Both *bd0816* and *bd3459* were not expressed during the free-swimming “attack phase” of *Bdellovibrio*, when it is outside prey, but showed strong expression peaks at 15 minutes post-infection which coincides with *Bdellovibrio* attachment to the prey cell and the start of invasion into the prey cell periplasm (Figure 1). At 30 minutes post-infection, *bd0816* and *bd3459* both showed a reduced expression level and expression was undetectable after 45 minutes (for *bd0816*) and 1 hour (for *bd3459*) post-infection. Thus *bd0816* and *bd3459* gene products are likely to be used for prey invasion, and the *bd3244* is likely to be used in a housekeeping role.

Expression patterns of *bd0816* and *bd3459* suggested that both may have a predatory role in facilitating *Bdellovibrio* entry into the bacterial prey periplasm and the formation of the rounded bdelloplast structure, in contrast to a likely housekeeping role for the constitutively expressed product encoded by *bd3244* (*dacB*) in maintaining the *Bdellovibrio's* own cell wall integrity. Thus we used deletion mutagenesis to further clarify any predatory roles of the Bd0816 and Bd3459 proteins. Both single and double deletion strains were constructed, and grown as both predatory and host-independent (HI) cultures. Growth measurement in triplicate over 72 hours showed that there was no significant difference in growth rates or morphologies for HI cultures of these mutants, further supporting the idea from the transcriptional studies that they have a predatory role.

### *E. coli* prey invaded by *bd0816* and *bd3459* mutant *Bdellovibrio* had altered bdelloplast morphologies versus those produced by wild type (WT)

**Figure 2:**
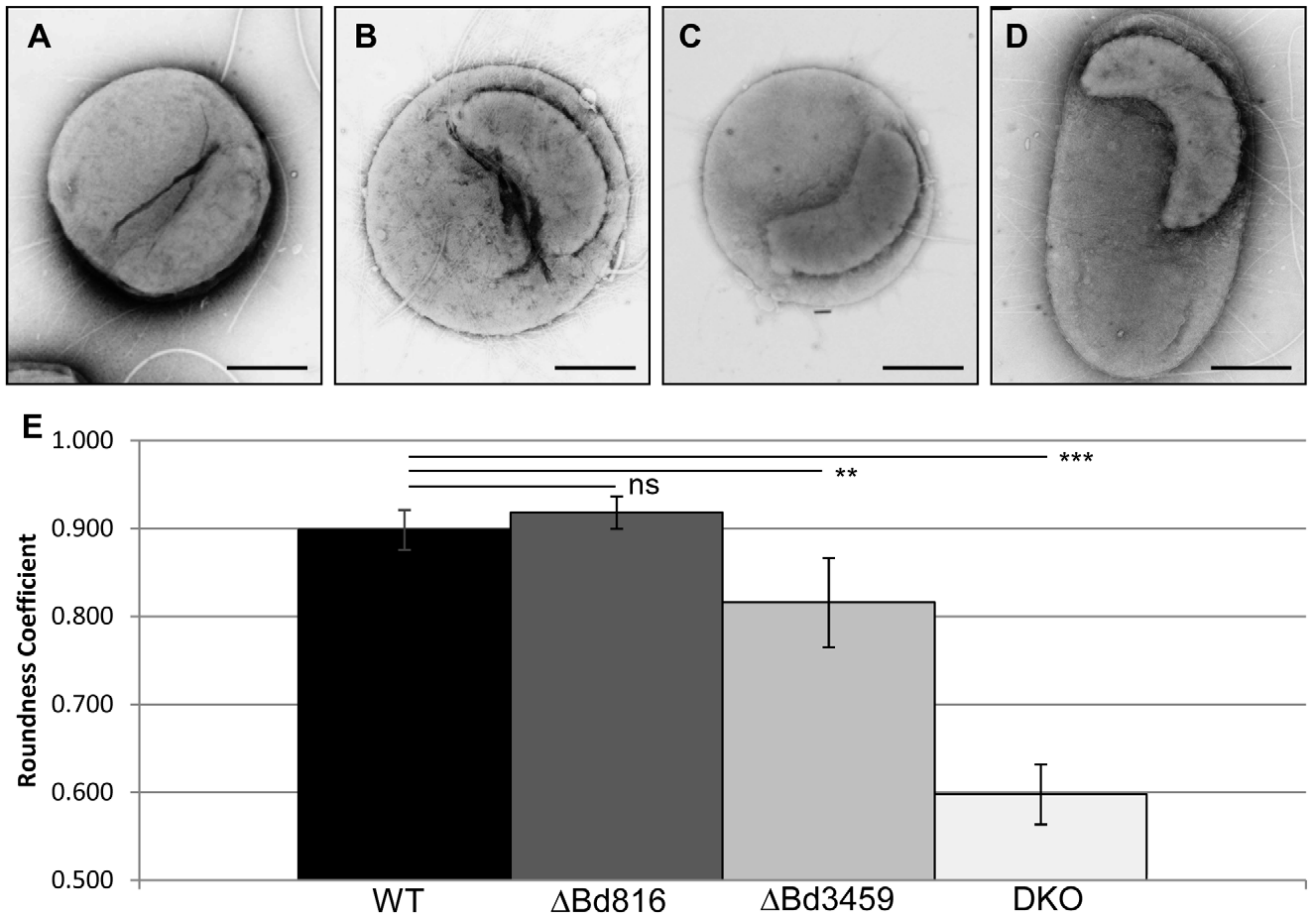
Electron micrographs and roundness analysis of wild type and *dacB* knockout-mutant infected *E. coli* prey cells. Images were taken 90-minutes post-invasion and the roundness of infected prey cells were analysed using ImageJ software. Invasion by **A**: Wild-type HD100 (100% of bdelloplasts were rounded, N = 25); **B**: HD100 Δ*bd0816* (100% of bdelloplasts were rounded, N = 21); **C**: HD100 Δb*d3459* (75% of bdelloplasts were rounded, N = 24); **D**: HD100 Δ*bd0816* Δ*bd3459* (DKO) double mutant (<10% bdelloplasts were rounded, N = 53). **E**: Bar graph showing the average roundness coefficient of the bdelloplasts from EM images with error bars showing 95% confidence intervals and statistical analysis of the means compared to WT (ns = not significant; ** = p<0.005; *** = p<0.001). Data is taken from 4 independent experiments. All displayed images were taken from the same experimental repeat at 80 kV (20,000-fold magnification) and stained with 1% PTA for 1 min. Scale bar is 500 µm.

Electron microscopy (EM) of typical bdelloplasts of *E. coli* invaded by wild type *B. bacteriovorus* HD100 (Figure 2A) compared to three gene-knockout mutant strains (Figure 2B Δ*bd0816*; Figure 2C Δ*bd3459*; Figure 2D Δ*bd0816* Δ*bd3459*), showed that all strains invaded prey but that the bdelloplasts formed had different shapes (larger fields of view can be seen in Figure S1). To investigate the differing bdelloplast shapes quantitatively, traces of the bdelloplasts in the EM images were made and measures of ellipticity were used (see Materials and Methods) to find an average ‘roundness’ coefficient for each invading *Bdellovibrio* strain (Figure 2E). Wild type HD100 *Bdellovibrio*-infected bdelloplasts were invariably rounded-spherical (N = 25; mean roundness = 0.899 (±0.058)). The same was true of the HD100 Δ*bd0816* mutant strain (N = 21; mean roundness = 0.918 (±0.043); p>0.05 Student's T-test compared to WT). The HD100 Δ*bd3459* mutant strain had a mixture of rounded bdelloplasts (75%) and more ovoid bdelloplasts (25%) contributing to a significantly reduced roundness coefficient (N = 24; mean roundness = 0.816 (±0.127); p<0.005 Student's T-test compared to WT); this is perhaps a result of inconsistency in the availability of Bd0816 protein to compensate (if it does) for the absence of Bd3459. The double Δ*bd0816 Δbd3459* mutant exhibited >90% of bdelloplasts which were clearly rod shaped (normal prey shaped) rather than showing the typical rounded bdelloplast morphology and the roundness coefficient was consequently highly significantly reduced (N = 53; mean roundness = 0.598 (±0.127); p<0.001 Student's T-test compared to WT).

These results suggested that the enzymes encoded by *bd0816* and *bd3459* were together responsible for effecting the characteristic rounded shape of the bdelloplast, and that Bd3459 may have a major effect in reshaping the prey cell, with Bd0816 contributing to this.

Throughout our study, it was not possible to complement mutants as plasmid complementation in *Bdellovibrio* requires conjugation from a donor *E. coli* strain (electroporation is not possible). Unfortunately, unregulated expression of *bd3459* killed the *E. coli* strains and there are no inducible/repressible vectors for *Bdellovibrio*. Thus, we used comparison of double to single mutants' phenotypes as the best substitute. That there is no gene downstream of *bd0816* in the same transcriptional orientation suggests that polar effects of the silent deletion of *bd0816* would not be expected. There is a single gene downstream of *bd3459* in the same transcriptional orientation, but this has homology to genes encoding non-enzymic products and would not be predicted to contribute to observed phenotypes.

Although bdelloplast morphology was grossly altered, the mutant *Bdellovibrio* did complete their predatory lifecycle. Viable plaque counts of the *Bdellovibrio* on prey lawns showed that although the emergence of the progeny *Bdellovibrio* from the prey was at 4 hours after invasion for the wild type HD100 and 5 hours for the double Δ*bd0816* Δ*bd3459* mutant strain, the yield of *Bdellovibrio* did not differ significantly, being a mean of 3.9 *Bdellovibrio* per prey cell for the wild type and 3.75 *Bdellovibrio* per prey cell for the double mutant. Thus in our experimental conditions, bdelloplast shape was not important for maximising progeny number. We then asked, because of the observed change in emergence time of the double mutant versus wild type, whether the activity of Bd3549 and Bd0816 might be to minimise the overall invasion time after the *Bdellovibrio* had attached to the prey.

### Wild type *B. bacteriovorus* HD100 invaded the prey periplasm more rapidly than the double Δ*bd0816* Δ*bd3459* mutant strain

**Figure 3:**
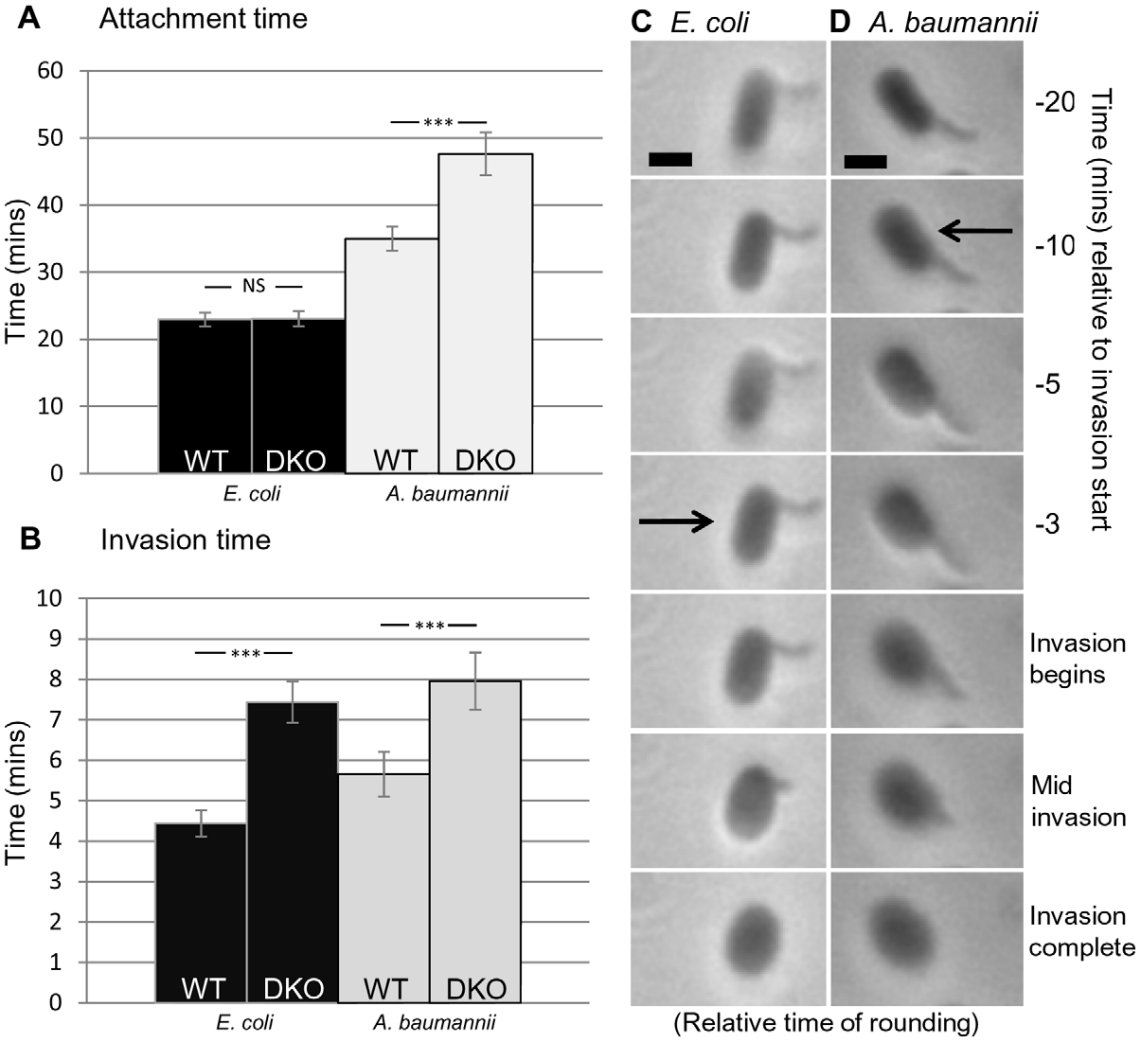
Histograms and images of *B. bacteriovorus* HD100 and Δ*bd0816* Δ*bd3459* strains infecting *E. coli* K-12 MG1655 and *A. baumannii*. Histograms of mean times for attachment (**A**) and invasion (**B**) by *B. bacteriovorus* HD100 wild type (WT) and Δ*bd0816* D*bd3459* (DKO) strains infecting *E. coli* K-12 MG1655 (black fill) and *A. baumannii* (grey fill); (Mean attachment time - as measured from initial *Bdellovibrio* contact with outside of prey cell to the start of traversal through the prey cell wall. Mean invasion time - as measured from the start of traversal through the prey cell wall to not being visible outside the prey cell, i.e. being completely within the prey cell) (N = 50 for all experiments). At least two independent experiments were carried out with error bars showing 95% confidence intervals and statistical analyses shown (ns = not significant; *** = p<0.001). Time-lapse images showing prey rounding relative to invasion for **C**) *E. coli* and **D**) *A. baumannii* by wild type HD100 *Bdellovibrio*. Arrowed box shows relative time at which rounding first becomes obvious. *E. coli* begins to round on average 2.3 minutes before invasion (N = 50), whereas *A. baumannii* begins to round on average 10.7 minutes before invasion (N = 50). *E. coli* finishes prey rounding concurrently with the completion of invasion, whereas *A. baumannii* tends to have its rounding completed earlier. Refer to results section for more information. Scale bar is 2 µm.

Time lapse phase contrast microscopy (examples in Videos S1, S2, S3, S4) measured frame-by-frame how long it took wild type *B. bacteriovorus* HD100 or the double mutant Δ*bd0816* Δ*bd3459* to completely pass through the prey cell outer membrane and form a bdelloplast. The overall invasion time of the wild type *Bdellovibrio* strain compared to the mutant strain was investigated in two different prey backgrounds. *E. coli* and *Acinetobacter* have a substantially different proportion of cross-linked peptides in their peptidoglycan (33% and 61% respectively) [15] and were chosen as prey species to investigate whether there is a link between the relative amount of time that the mutant strain takes to invade compared to wild type within prey cells where the extent of cross-linking of the peptidoglycan differs (to test whether the mutant cannot hydrolyse the cross-links as efficiently). Measurements of how long a single *Bdellovibrio* was attached to a prey cell were averaged (attachment: Figure 3A), as well as measurements of how long it took for the *Bdellovibrio* to actually pass through the prey cell wall into the periplasm (invasion: Figure 3B); frequency distributions are included in Figures S2, S3, S4, S5. Double deletion of *bd0816* and *bd3459* resulted in a significantly increased overall invasion time (11.5% longer for *E. coli* prey (WT: 27.4 mins (±4.04); Δ*bd0816* Δ*bd3459*: 30.54 mins (±4.40); p<0.001 Student's T test) and 36.7% longer for *A. baumannii* prey (WT: 40.66 mins (±6.98); Δ*bd0816* Δ*bd3459*: 55.6 mins (±12.63); p<0.001 Student's T test)); frequency distributions are included in Figure S6 and S7. Therefore one function of the predatory PBP4 proteins is to allow *Bdellovibrio* to invade prey faster, particularly when invading prey with a more highly cross-linked cell wall. Interestingly, *E. coli* attachment time (Figure 3A) was not significantly different between the *Bdellovibrio* strains, whereas the invasion time (Figure 3B) was (WT: 4.44 mins (±1.18); Δ*bd0816* Δ*bd3459* mutant: 7.44 mins (±1.82); p<0.001 Student's T test). For *A. baumannii*, the mean attachment and invasion times were significantly different between the two *Bdellovibrio* strains: (WT attachment: 35.0 mins (±6.51); Δ*bd0816* Δ*bd3459* mutant attachment: 47.64 mins (±11.52); p<0.001 Student's T test) (WT invasion: 5.66 mins (±1.99); Δ*bd0816* Δ*bd3459* mutant invasion: 7.96 mins (±2.56); p<0.001 Student's T test).

There was no significant difference in the cell lengths of the wild type and mutant *Bdellovibrio* strains. Therefore we can be confident that the observed differences in invasion times are not affected by some *Bdellovibrio* having longer cell bodies to insert into prey. Overall *Bdellovibrio* invasion success rates were not affected in the mutant with more than 97% of attempted prey invasions being successful in all strains (versus 3% of attachments where the *Bdellovibrio* abandoned the prey surface); so the Bd3459 and Bd0816 proteins are not absolutely required for invasion *per se* but speed the process. The evolutionary significance of this is discussed later.

### Timing of the rounding of prey cells during invasion by wild type *Bdellovibrio*

In the gallery of frame by frame stills (Figure 3C) showing *Bdellovibrio* invasion into *E. coli*, the prey cell began rounding just prior (mean 2.3 min) to the first sign of *Bdellovibrio* entry into the periplasm, and the rounding process was completed concurrently with the *Bdellovibrio* completing entry. In contrast, for invasions into *A. baumannii* (Figure 3D), the rounding began earlier relative to invasion (mean 10.7 min, but up to 26 minutes) and the rounding process was completed on average 4.1 minutes before the *Bdellovibrio* had completed its entry. In no cases had the wild type *Bdellovibrio* passed into the periplasm of the prey cell before rounding the prey cell up. In summary, in the more highly peptidoglycan-cross-linked environment of the *Acinetobacter* [15], the cell became rounded at a noticeably earlier time relative to invasion than *E. coli*, despite the overall invasion time being longer. We observed that *Acinetobacter* (Video S5) becomes more malleable than *E. coli* once invaded and deforms when in contact with another cell whereas invaded *E. coli* do not (Video S6). This also shows that peptidoglycan de-cross-linking (due to endopeptidase activity) is not localised to the area that the *Bdellovibrio* is in contact with, as the opposite ‘non-invaded’ side of the *Acinetobacter* prey cell also became deformed.

### Bd0816 and Bd3459 activity reduced the frequency of double invasions of single prey

**Figure 4:**
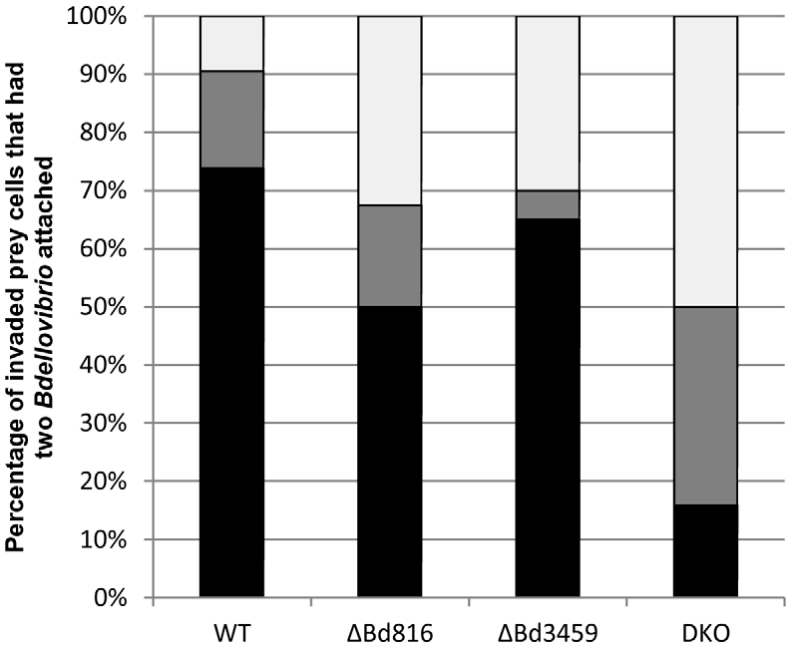
Frequency of double invasions of *E. coli* prey during attack by *B. bacteriovorus* HD100 wild type, HD100 Δ*bd0816*, HD100 Δ*bd3459* or *B. bacteriovorus* HD100 Δ*bd0816* Δ*bd3459* (DKO) strains. Time-lapse microscopy was used to observe invasions when two *Bdellovibrio* cells were seen to attach to a single prey cell for longer than 10 minutes (longer than the usual ‘recognition’ period) and was taken to be an ‘attempted invasion’ by both cells. The outcomes of invasions (N = 42 for HD100 WT, N = 40 for both HD100 Δ*bd0816* and HD100 *Δbd3459*, N = 44 for HD100 Δ*bd0816* Δ*bd3459*) were tallied into one of three categories; i) ‘single infection’ (black bars; just one *Bdellovibrio* successfully invades); ii) ‘synchronous infection’ (grey bars; both invade successfully at the same time); or iii) ‘tailgating infection’ (white bars; one invades followed by the other). The double mutant *Bdellovibrio* strain showed a much higher proportion of double invasions and therefore decreased predatory population fitness due to self competition in single prey. The absence of Bd3459 has less of an effect on double invasions than the absence of Bd0816 although both single mutant datasets show an intermediate effect between WT and the double mutant. Data were taken from at least two experimental repeats. See Materials and Methods for more information.

After invasion, *Bdellovibrio* uses the prey cell contents to produce the maximum progeny possible from the limited prey resources [16]. Thus predatory fitness is lowered if more than one *Bdellovibrio* invades an individual prey cell, as this would introduce intra-species competition and reduce the available pool of *Bdellovibrio* in the environment to invade other prey cells; double invasions are indeed rare for WT *Bdellovibrio* [17]. We noticed in our time lapse microscopy experiments that the Δ*bd0816* Δ*bd3459* double mutant *Bdellovibrio* showed a much higher proportion of double invasions than wild type, and we quantified this by microscopy (Figure 4) for cases where there were two *Bdellovibrio* cells attached to the outside of a single uninfected *E. coli* prey cell. We also investigated this (Figure 4) in the Δ*bd0816* and *Δbd3459* single mutant strains. The outcomes of invasions (N = 42 for WT, N = 40 for single mutants, N = 44 for double mutant) were categorised (examples in Videos S7, S8, S9, S10, S11, S12) into one of three categories: i) single invasion; ii) synchronous double invasion or iii) tailgating double invasion where one *Bdellovibrio* had entered the prey before the second entered. In 73.8% of wild type invasions, only one *Bdellovibrio* (from two attached) entered prey, whereas this proportion dropped to 15.9% in the double mutant; the single mutants showed intermediate results between wild type and the double mutant (Δ*bd0816* had 50% and *Δbd3459* had 65% of such ‘single’ invasions) (Figure 4). The proportion of synchronous invasions was 16.7% for the WT strain, 17.5% for the Δ*bd0816* strain, 5% for the *Δbd3459* strain, and 34.1% for the double mutant strain. Finally, only 9.5% of WT *Bdellovibrio* invasions were tailgating whereas 50% were for the double mutant; again the single mutants showed intermediate results (Δ*bd0816* had 32.5% and *Δbd3459* had 30% of such ‘tailgating’ invasions). We used Fisher's Exact Tests to look at the significance of these differences (GraphPad InStat). This revealed that the single and double mutants had a significantly higher occurrence of tailgating invasions than wild type (WT vs. Δ*bd0816* p = 0.0102 (*); WT vs. Δ*bd3459* p = 0.0188 (*); WT vs. Δ*bd0816* Δ*bd3459* p<0.0001 (****)). Of the 9.5% of the rare tailgating WT *Bdellovibrio*, the inter-invasion time gap (time between the first cell entering fully and the second cell entering) ranged from 0 to 5 minutes (mean 1.25 min), whereas the double mutant had inter-invasion time gaps ranging from 0 to 13 minutes (mean 4.1 min).

### Heterologous overexpression of active Bd3459 protein in *E. coli* caused cell shape changes and lysis in hypotonic media

**Figure 5:**
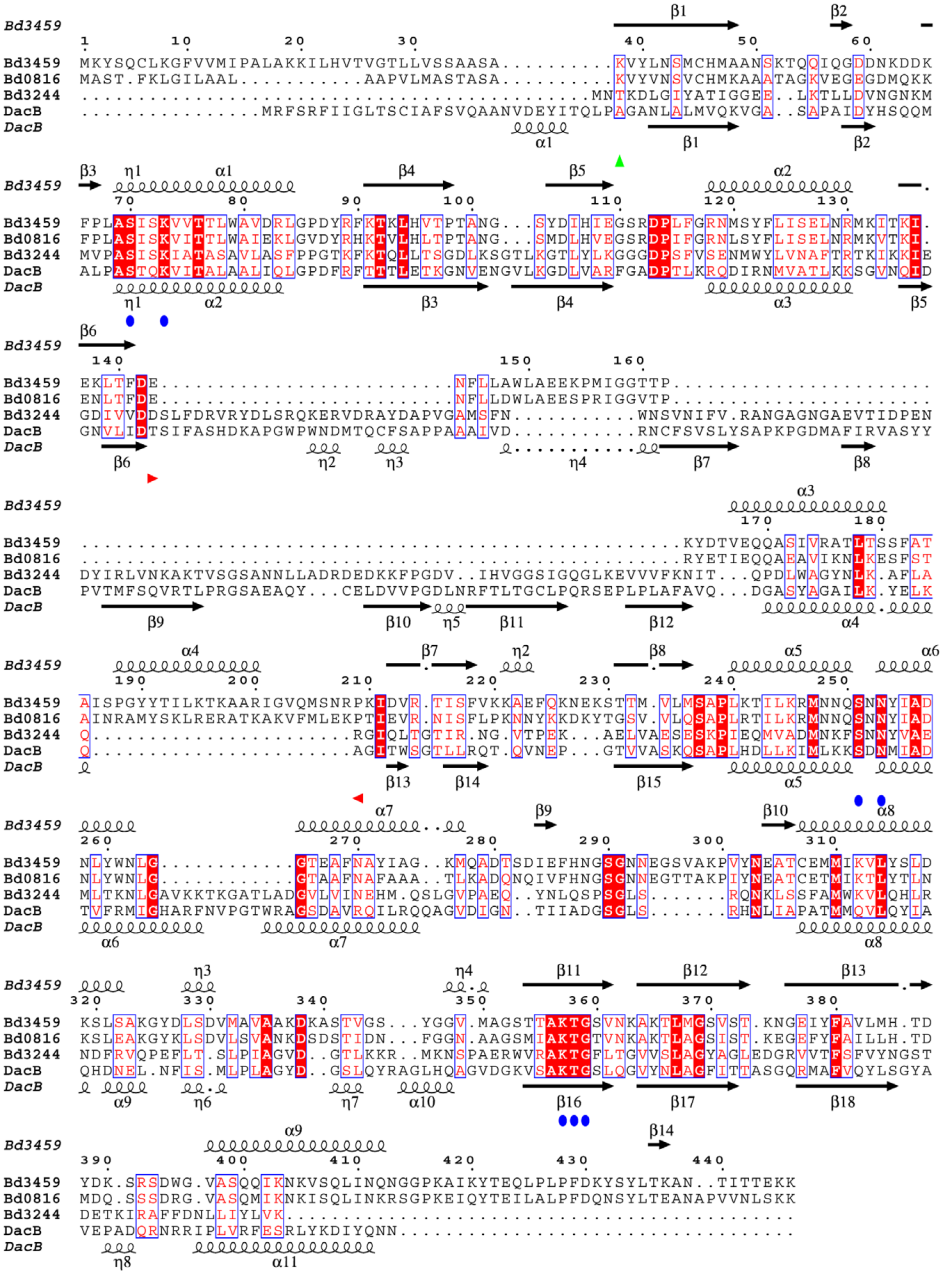
Alignment of *Bdellovibrio* “housekeeping” Bd3244 and predatory Bd0816/Bd3549 proteins versus housekeeping PBP4 of *E. coli*. The structural elements of the *E. coli* PBP4 and the Bd3459 proteins are displayed respectively below and above the alignment. The central structural differences between Bd3459/Bd0816 PBP4s and *E. coli* PBP4/Bd3244 (reflected in missing/altered domains in the structure (see Figure 8) can be clearly seen.

Overexpression of Bd3459 in *E. coli* TOP10 was used to test the hypothesis that Bd3459 was directly responsible for rounding up of the prey cell due to peptidoglycan modification. The native *B. bacteriovorus* HD100 *bd3459* gene, including signal sequence to permit periplasmic export (if recognised by *E. coli*), was expressed from the Invitrogen pBADHisA expression vector (which had its native His-tag removed), under the control of the *araBAD* promoter which is inducible by L-arabinose and further repressible by glucose. As a control and to link into the structural information (below), a Bd3459 S70A site-directed variant was made in which the conserved PBP active site serine (Figure 5) was substituted in a way that would be predicted (from general PBP structural knowledge [11]) to produce a functionally-inactive PBP4 protein. SDS-PAGE of periplasmic extracts from *E. coli* expressing the Bd3459 WT and S70A protein showed comparable intensities of Coomassie-blue stained induced protein bands of 46 kDa (Figure S8) confirming that equivalent quantities of each protein were exported to the periplasm.

**Figure 6:**
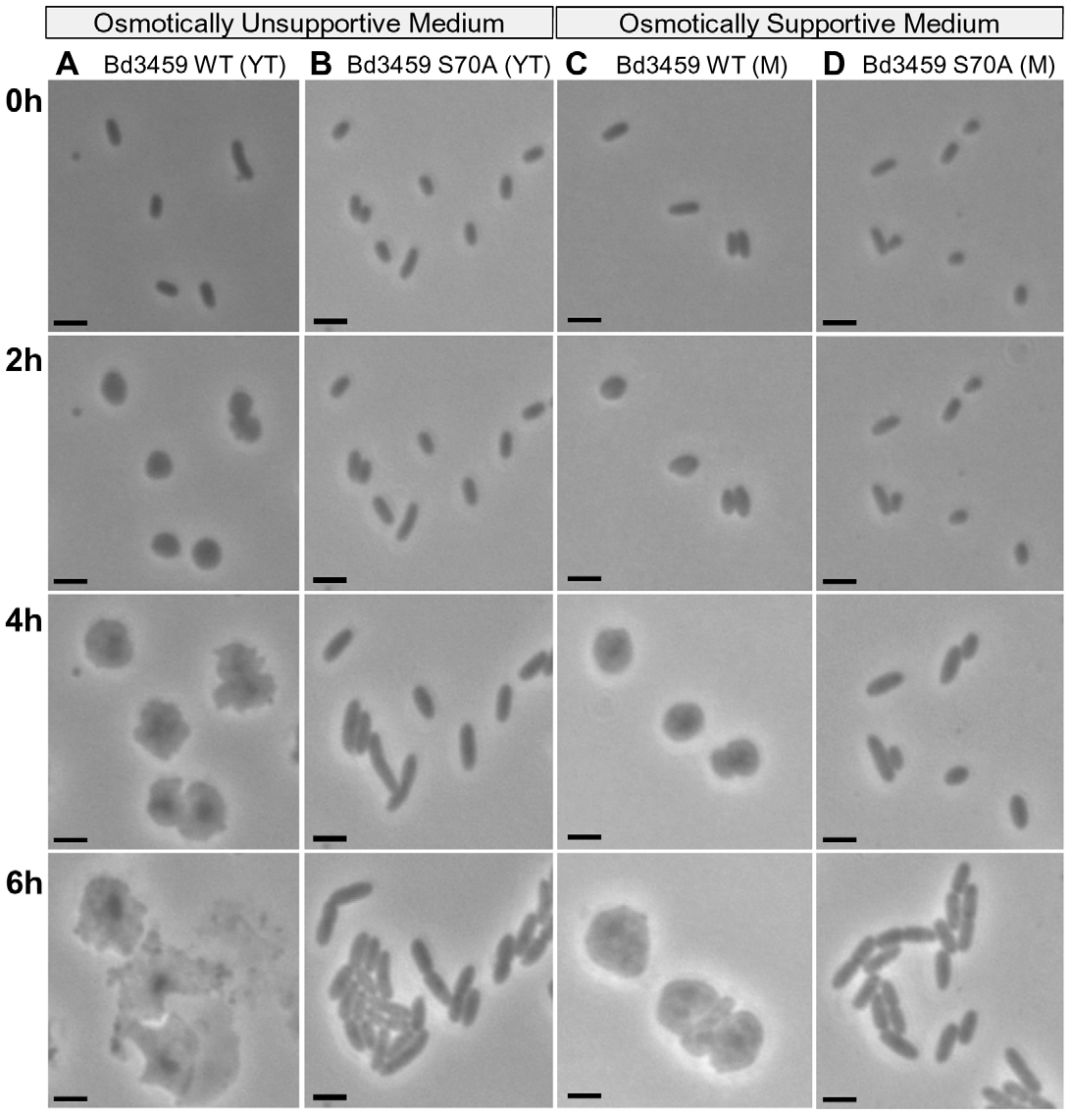
Expression of full-length Bd3459 protein alone, but not a S70A active site variant, causes rounding/lysis of *E. coli*. Time-lapse microscopy of *E. coli* TOP10 cells containing either wild type (WT) or active site variant (S70A) Bd3459 protein being overexpressed from an L-arabinose inducible plasmid by addition of arabinose at 0 hours. Cells were immobilised on a YT-agarose pad (YT) or osmotically stabilised M-medium-agarose pad (M), containing 0.2% (v/v) L-arabinose and development over 6 hours is shown. A = Bd3459 (WT) on YT; B = Bd3459 (S70A) on YT; C = Bd3459 (WT) on M; D = Bd3459 (S70A) on M. Osmotically unsupported *E. coli* expressing WT Bd3459 lyse and osmotically supported *E. coli* round up. *E. coli* expressing the S70A protein variant continues to grow normally. Also see Videos S13, S14, S15, S16.

Expression of the WT Bd3459 protein caused dramatic morphological changes in the expressing *E. coli* TOP10 strain (Figure 6) but these were not seen when the S70A mutant protein was induced. Time-lapse microscopy of these Bd3459-expressing *E. coli* immobilised on a YT-agarose pad containing arabinose (Figure 6A and 6B, Video S13 and S14) showed that during the first hour, the cells expressing WT and S70A Bd3459 grew slightly but by 2 hours, distinct rounding and localised blebbing were visible in the *E. coli* expressing wild type Bd3459; however there was normal growth and septation of the *E. coli* expressing the S70A Bd3459. For cells expressing WT Bd3459, the blebs continued to expand until the whole *E. coli* diameter had increased and formed cells with differing irregular shapes. From 4 hours onwards, the *E. coli* cell diameter increased dramatically until eventually cell lysis occurred between 6–8 hours after arabinose induction of WT Bd3459 (Figure 6A). The overexpression of the S70A variant of Bd3459 throughout caused no abnormal cell wall defects and the *E. coli* grew by binary fission (Figure 6B).

The experiments expressing Bd3459 were repeated (Figure 6C and 6D, Video S15 and S16) in sufficiently hypertonic medium (M-medium, including 0.23 M sucrose [18]) to try to osmotically support the damaged *E. coli* cells and prevent the movement of water into them which we hypothesised was causing cell lysis; this is usually resisted by the intact cell wall. During overexpression of WT and S70A Bd3459 in *E. coli* on this M medium, *E. coli* cells with the S70A Bd3459 protein replicated slowly, but in a normal binary fission manner (Figure 6D). Cells expressing the WT Bd3459 protein failed to grow or septate, and instead spheroplasted after 1–3 hours into structures reminiscent of bdelloplasts (Figure 3C). After 4 hours, these structures slowly increased in diameter, and retained a markedly more spherical shape compared to the cells grown on YT medium (Figure 6A). Eventually the cells lysed, whether this was due to dehydration of the pad affecting the tonicity of the medium or another effect remains unknown.

### *In vitro* activity of Bd3459

**Figure 7:**
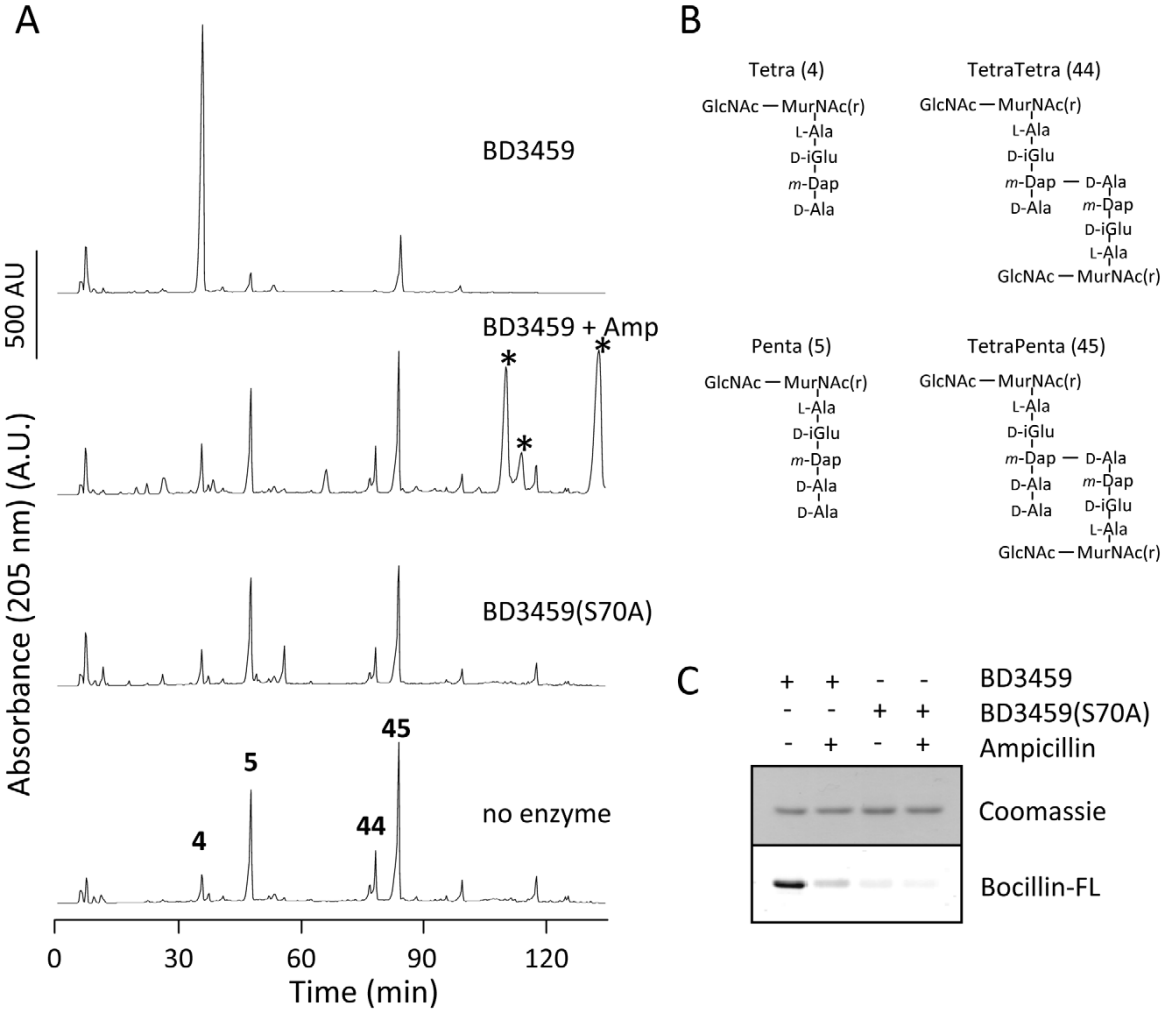
Activity of Bd3459 and its inhibition by ampicillin. (**A**) Pentapeptide-rich peptidoglcyan was incubated with Bd3459, Bd3459 pre-incubated with ampicillin, Bd3459 (S70A) or no enzyme, followed by digestion with cellosyl, reduction with sodium borohydride and analysis of the resulting muropeptides by HPLC. Muropeptides: 4, Tetra; 5, Penta; 44, TetraTetra; 45, TetraPenta. Peaks originating from ampicillin are indicated by a star. Bd3459, but not Bd3459 (S70A) or ampicillin-blocked Bd3459, digested Penta and TetraPenta to Tetra indicative of a DD-endo/carboxypeptidase activity. (**B**) Structures of the reduced muropeptides. GlcNAc, N-acetylglucosamine; MurNAc(r), N-acetylmuramitol; L-Ala, L-alanine; D-iGlu, D-isoglutamic acid; *m*-Dap, *meso*-diaminopimelic acid; D-Ala, D-alanine. (**C**) Bd3459 and Bd3459 (S70A) were incubated with or without ampicillin, followed by labelling with Bocillin-FL and SDS-polyacrylamide gel electrophoresis. Bocillin-FL was detected by fluorescence (Bocillin-FL); total protein was visualized by staining with Coomassie Blue. Bocillin-FL-binding of Bd3459 was greatly inhibited by pre-incubation with ampicillin; Bd3459 (S70A) bound substantially less Bocillin-FL than Bd3459. Thus, Bd3459 is a penicillin-binding protein and degrades isolated cell wall material.

Purified Bd3459 (without signal sequence) was able to significantly reduce the amount of cross-linked and uncross-linked pentapeptides when incubated with peptidoglycan from a pentapeptide-rich *E. coli* strain (Figure 7A and 7B), yielding mainly the un-crosslinked tetrapeptides. The addition of the beta-lactam ampicillin inhibited the WT enzyme, and the Bd3459 S70A version of the enzyme was always inactive (Figure 7A and 7B). Bd3459 covalently bound the fluorescent beta-lactam Bocillin-FL, a reaction that was strongly inhibited by pre-incubation with ampicillin (Figure 7C). Compared to the active enzyme, Bd3459 (S70A) bound significantly less Bocillin-FL as would be predicted. Together these data establish that Bd3459 is a PBP with DD-carboxy- and DD-endopeptidase activity, as suggested by the bioinformatic analysis of the protein sequence (Figure 5).

### The Bd3459 PBP structure has several adaptations that differ from “housekeeping” PBP4-type enzymes

**Figure 8:**
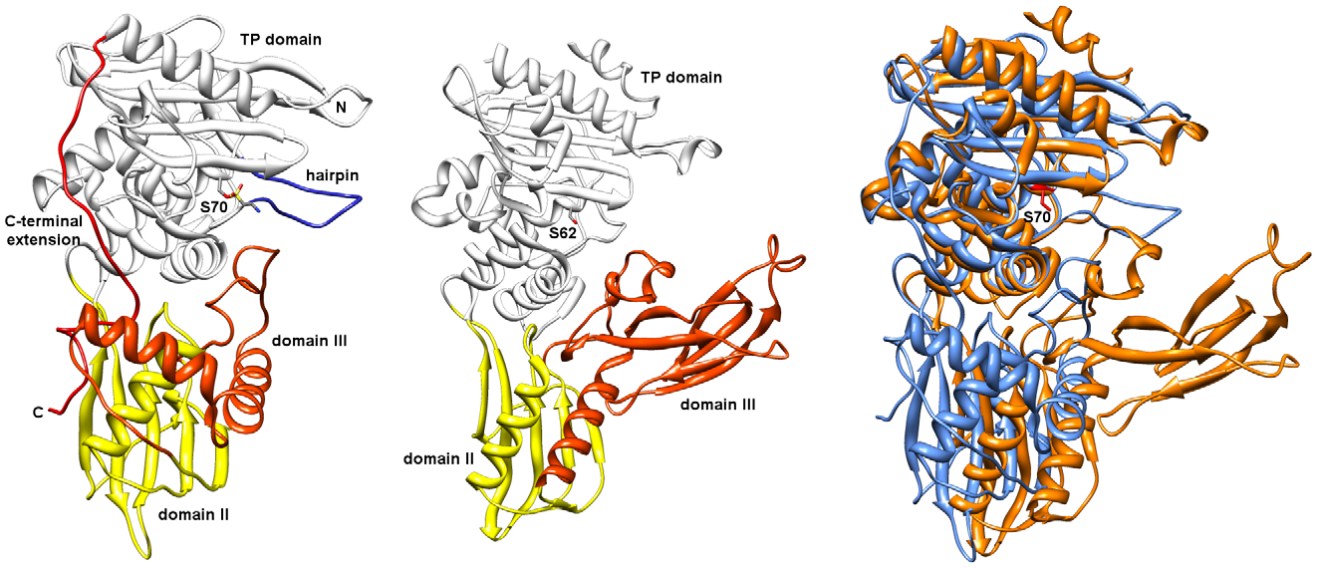
Crystal structure of *B. bacteriovorus* Bd3459 revealing predatory adaptations differing from the “PG-housekeeping” PBP4 of *E. coli*. Bd3459 left hand panel, *E. coli* PBP4 centre panel, coloured thus: TP domain, white; domain II, yellow; domain III, orange; “insert” hairpin, purple; C-terminal “staple”, red. The active site serine residues of Bd3459 (S70 sulfonyl adduct) and *E. coli* PBP4 (S62) are shown in stick form. Domain III of *E. coli* PBP4 acts to functionally constrict the active site (for a more attenuated/regulated self-peptidoglycan metabolism), whereas Bd3459 lacks this and thus may act promiscuously on a range of larger peptidoglycan substrates. This would fit with the predatory role of Bd3459 shown genetically. Right hand panel is composed of Bd3459 (blue, with S70 coloured in red) and *E. coli* PBP4 (orange) superimposed via their transpeptidase domains, illustrating the relatively more solvent-exposed active site of Bd3459 and respective differences in domains II and III.

We solved the high-resolution structure of the predicted secreted form of Bd3459 (AA38-446, solved to 1.45 Å resolution, SIRAS phasing from a platinum derivative), the general fold of which is shown in Figure 8 (co-ordinates and structure factors have been deposited in the RCSB, accession code 3V39). The relatively high resolution of the structure is in contrast to the high solvent content (69%), which results from Bd3459 packing in a tubular fashion around the c-axis and forming large “pores” in the crystal. The nature of this packing indicates that unlike certain PBP4 proteins [11], Bd3459 is likely to be monomeric in solution. Bd3459 is comprised of three domains, two of which are found in other PBP4 proteins, the classical “TP” transpeptidase domain (domain I; K38-D87 and P239-L427), and an associated domain (domain II; Y88-A238 and P428-C terminus). This modularity is reflected in structural similarity searches with DALI [19], which can be split into two groups – the highest sharing both domains (Z-scores 20–30, PBP4-subfamily, including the R39 DD-peptidase from *Actinomadura* sp. R39, PBP4a from *B. subtilis*, and PBP4 from *E. coli* and *H. influenzae*), and lower-scoring results matching the TP domain only (Z-scores 20 and below, low MWt PBP TPases and β-lactamases, *e.g.* PBP4 from *Staphylococcus aureus*, PBP3 from *Streptococcus pneumoniae* and SHV-1 from *Klebsiella pneumoniae*).

Aside from this close match to PBP4-like proteins, Bd3459 has several gross structural features that differ from this family, and that are presumably related to both its secretion and predatory functions. These are highlighted on the alignment comparing housekeeping PBP4 sequences to Bd3459 and Bd0816 (Figure 5) and are very evident in the crystal structure (Figure 8). Firstly, there is an unusual N-terminal turn - the start of our construct, M37 (expression construct N-terminal methionine) sits in a turn formed by the final α-helix of the TP fold. It is apparent that in the mature, secreted form (whose N-terminal residue will be K38) the space occupied by the M37 sidechain may be taken by other residues (e.g. W395, which is in two weakly observable rotamer forms, and has presumably flipped out of the pocket to accommodate the introduced methionine). A disulphide bond between residues C45 and C307 stabilizes the interaction between the N- and C-terminal regions of the TP domain, and is conserved in Bd0816 (Figure 5). A long C-terminal extension to the TP domain (residues G414 onward) threads back through the fold, eventually completing the β-sheet of domain II; this element is not observed in other TP structures and presumably acts to stabilize the protein (residues I419, Y421, P428, F429, Y434 and L435 pack against the hydrophobic core of both domains). Residues 439–446 have weak density and were left unmodelled at the very C-terminus.

**Figure 9:**
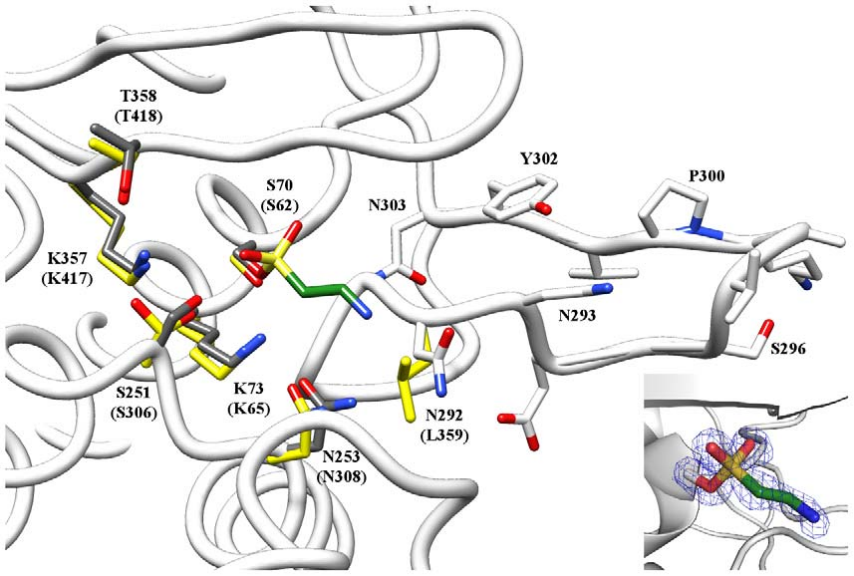
Structural comparison detail of overlapping active site residues (SXXK, SXN, KTG) and extra “hairpin” domain of Bd3459 versus *E. coli* “housekeeping” PBP4 protein. Comparison of *E. coli* PBP4 (coloured yellow, active site serine S62, only selected residues shown) and Bd3459 (coloured white, active site serine S70) active sites. The additional hairpin domain of Bd3459 is shown from N292–N303. Despite the more accessible conformation shown in Figure 8, Bd3459 retains the conserved catalytic apparatus of PBP4 enzymes, in a productive (predicted active) conformation (tallying with the activity assays, and observance of a covalent adduct). Insert (right) shows the refined density for the sulphonyl linkage to S70 (1.2σ, 1.45 Å resolution).

The largest structural difference in Bd3459 in comparison to the PBP4-grouping of enzymes is the absence from Bd3459 of a classical domain III, an all-beta structure that sits adjacent to the TP active site in conventional PBP4s and is responsible for PBP4 dimerization [11]. Domain III has also been postulated to regulate substrate access and limit the ability of PBP4 proteins to bind bulky substrates [20]. It is of interest (and relevance to the wide diversity of bacterial prey walls that *Bdellovibrio* attack) that Bd3459 possesses no compensatory regions in this part of the fold, leading to a much more accessible active site (Figures 8 and 9). The domain II:III:TP contacts of housekeeping PBP4 enzymes are replaced in Bd3459 by a region of novel structure (Figures 8 and 9), composed of AA 144–210. This region contains a small helix with irregular geometry (AA 153–159) that packs against the TP SXN motif, and two standard helices (α3, α4) that wrap around the β-sheet of domain II. The α4 helix contacts the TP C-terminal extension, obscuring α2 which is usually solvent-exposed in PBP4 proteins.

### Active site architecture and Ser70 covalent modification of Bd3459

The SXXK, SXN and KT/SG motifs are invariant among TP structures, conserved between transpeptidase, endopeptidase and carboxypeptidase functions [5]. These residues are present in Bd3459 and Bd0816 (Figure 5), and adopt what would be predicted to be an active conformation in our Bd3459 structure (Figures 8 and 9); residues S^70^ISK, S^251^NN and K^357^TG overlaying with their PBP4 homologues (in both apoenzyme and reacted forms), [9]–[11], [20], [21]. When building and refining the Bd3459 structure, it was apparent that a covalent modification of S70 had occurred during purification/crystallization. The excellent electron density indicated that the adduct was tetrahedral in nature and larger than what could be attributed to a simple phosphorylation. Initially, this modification was designated as originating from protease inhibitor inclusion (sulphonyl fluoride-based), but repurification and crystallization in the absence of inhibitor resulted in an identical adduct. After being unable to crystallize the protein when HEPES/MES was excluded, we have tentatively modelled the adduct as a covalently attached HEPES molecule, with disorder distal to the C9/C10 atoms. This type of reaction has precedent in the literature, having been observed for the functionally homologous tricorn proteases [22], and the structurally homologous carboxylesterases [23]. Indeed, sulphonyl analogues of β-lactams have been shown to react with the highly-homologous beta-lactamase family [24]. Such a reaction with a buffer molecule with a relatively poor leaving group (H_2_O) would indicate a high reactivity of S70. The tetrahedral arrangement of the reacted sulphonyl group is reminiscent of a transpeptidase acylenzyme transition state (e.g. [11]), with atom O1 occupying the classical “oxyanion hole” formed by the backbone amide groups of S70 and S360.

An insertion at the end of the β-strand carrying the KTG motif is observed for both Bd3459 and Bd0816 proteins (Figure 5). When comparing the Bd3459 and PBP4 structures, this sequence insertion takes effect between N292 and N303 and forms a “thumb-like” hairpin on the periphery of the active site pocket (Figure 9). Residue N292 substitutes for what is usually a hydrophobic residue in TP enzymes (e.g. L359 of *E. coli* PBP4), a residue that forms part of a pocket that binds the penultimate D-Ala of the peptide stem [11]. This substitution may reflect an altered substrate specificity for this pocket (unlikely given the conservation of pocket volume), or may be analogous to asparagine residues in β-lactamases that orient water molecules for purposes of enzyme deacylation [25]. This latter possibility would fit well for a hydrolytic function of Bd3459. Residue N293 anchors the base of the hairpin, forming unusual bifurcated hydrogen bonds to the backbone amides of G295 and V301. The positioning of the hairpin is similar to the Ω-loop of class A β-lactamases (involved in deacylation, [26]) or the Ω-like loop of PBP3 (involved in substrate position 3 Lys/*meso*-Dap discrimination, [27]), but is structurally very different from both.

In comparison to PBP4 proteins, the Bd3459 hairpin takes the place of a region that closes off the active site cleft and is responsible for forming a pocket to bind a non-cross-linked *meso*-Dap sidechain (e.g. D155, F160 and R361 of *E. coli* PBP4 [28]). Strikingly, Bd3459/Bd0816 possess no counterparts to these residues, and presumably do not bind *meso*-DAP termini in such a fashion. Comparison with the peptidoglycan-mimetic complex of *B. subtilis* PBP4a [10] suggests that the hairpin region of Bd3459 would be ideally placed to recognize a cross-linked *meso*-Dap peptide, endowing specificity for higher order oligomers (see below). Such an arrangement would place a cross-link to be hydrolyzed directly at the active site and a second cross-link over the face of the hairpin (the peptidoglycan form known classically as a trimer [2], with potential tetramer linkage at the D-Ala following the *meso*-Dap of the hydrolyzed bond). Additionally, the presence of a threonine at position T342 is conserved between Bd3459/Bd0816 and PBP4 proteins, and correlates with active site accessibility, favouring endopeptidase over carboxypeptidase activity [10].

Given the link between peptidoglycan integrity and cell structure [6], [29], we propose that Bd3459 (and Bd0816 which is predicted to be structurally similar) function to hydrolyse cross-links in the prey's peptidoglycan in such way that an osmotically stable intact peptidoglycan is maintained while cell shape is altered; observations have been made of peptidoglycan remaining throughout the predatory life cycle by electron microscopy [13], [30]. This activity would be functionally converse to the *E. coli* transpeptidase PBP2, whose function is to synthesize cross-links to enable the cell to grow with rod-shape [31]. From a homology viewpoint, conventional housekeeping PBP4 proteins have been shown to have both DD-carboxypeptidase and DD-endopeptidase activities *in vitro*, but are likely to function solely as endopeptidases *in vivo* [32].

## Discussion

We have shown that *Bdellovibrio* express two related PBP4-like proteins Bd3459 and Bd0816, evolutionarily adapted for predation, which act on the prey peptidoglycan of diverse bacterial prey at the invasion stage of predation speeding the process and reducing self-competitive, double prey-entry events. Transcription of both genes (Figure 1) is short-lived peaking at 15 minutes into the predatory life cycle. As predatory growth proceeds by a series of prey-hydrolytic and predator-developmental processes, it is likely that the gene products are regulated post-transcriptionally so that their lytic activity is appropriately controlled. This may be by targeted PBP4 protein degradation or by specific inhibitors but determining this is beyond the scope of our study.

The action of the predatory PBP4s is seen as the rounding of the prey cell prior to *Bdellovibrio* entry (Figure 3C and 3D) making an osmotically stable bdelloplast structure but with more deformable peptidoglycan than the original prey cell. Previous studies [33] had considered that the increased intracellular space that prey rounding provides allows *Bdellovibrio* to overcome space constraints and maximise the yield of progeny *Bdellovibrio* per prey invasion. For *E. coli* prey we did not find this to be true, (although it may be more important in smaller sized prey) and this infers that prey rounding may be an unintentional by-product of the action of these enzymes which confer other predatory fitness advantages. Indeed, we have shown that the action of the Bd3459 and Bd0816 enzymes enable significantly faster invasions by WT *Bdellovibrio* than a double deletion mutant, particularly in prey with a higher proportion of peptidoglycan cross-links (*e.g. Acinetobacter*). These enzymes, each and together also inhibit self-competitive “tailgating” prey invasions, further increasing the *Bdellovibrio* predatory population fitness.

We consider that the peptidoglycan-de-cross-linking, endopeptidase action of the Bd3459 and Bd0816 enzymes may be important for one or both of the following reasons:

1. Reduction of the torsional stress of the prey cell wall pushing out on the *Bdellovibrio* as it tries to enter the periplasm- making it quicker to enter prey, and reach the inner membrane of the prey and kill it; thus the least time possible is spent by the *Bdellovibrio* as a “sitting duck” attached to a live prey bacterium which could swim away with it to a non-favourable environment. Because of the de-cross-linking activity of Bd3459 and Bd0816 the *Bdellovibrio* rapidly enters the prey cell and kills it and then has environmental protection from being inside it. The differing proportion of peptidoglycan cross-linking that the *E. coli* (33%) and *Acinetobacter* (61%) possess [15] may be a contributing reason why there are differing invasion times (Figure 3) between the two strains. Although other factors such as the presence of a capsule or other peptidoglycan differences may be responsible for this, one hypothesis is that in *Acinetobacter*, more de-cross-linking and therefore time would have to occur to achieve a minimum threshold through which the invading *Bdellovibrio* can fit. When the *Bdellovibrio* are attached to the outside of prey (by the *Bdellovibrio* non-flagellar pole) they are secreting the Bd3459 and Bd0816 enzymes to assist with prey cell wall modification for entry.
2. If the first *Bdellovibrio* invading a prey cell makes its peptidoglycan more “flaccid” and deformable (Video S5), by peptidoglycan de-cross-linking due to due to predatory PBP4-like protein action, as it enters it; then subsequently arriving *Bdellovibrio* find the prey wall softened. Atomic force microscopy studies on bdelloplasts versus prey cells [34] support this idea, showing that the spring constant of uninvaded *E. coli* prey cells was 3 times stiffer than that of bdelloplasts. As leverage inward by retraction of type IV pili bound to prey peptidoglycan is suggested as a predatory entry mechanism [35], [36], pili retracting against a less rigid, de-cross-linked prey cell wall (after action of the Bd3459 and Bd0816 enzymes) may be less successful and therefore the second *Bdellovibrio* will fail to enter. This prevents two *Bdellovibrio* competing for the same finite nutritional resource in a prey single cell and increases the fitness of the population.

The results from our experiments show that neither of the Bd0816 or Bd3459 proteins are required for prey entry itself. Previous studies [13] predicted that endopeptidase activity would not be required to make the pore in the peptidoglycan through which the *Bdellovibrio* invades. Those experiments predicted that this role would be carried out by a carefully regulated lytic transglycosylase. Transcriptional studies from our lab [14] showed that *bd3575*, encoding a lytic transglycosylase, is highly upregulated upon *Bdellovibrio* invasion. Deletion of Bd3575 still allowed invasion to occur, so it is possible that there is either another lytic transglycosylase to be found, another mechanism completely is responsible, or it is a combination of Bd3575, Bd0816 and Bd3459 that enable invasions; in this case a triple mutant may be required to abolish invasion.

In the Δ*bd0816* Δ*bd3459* mutant, we hypothesise that the cell wall remains rigid for longer and allows secondary invasions mediated by pilus retraction. In this case of extra bdelloplast wall rigidity, how might there be enough room for the Δ*bd0816* Δ*bd3459* mutant *Bdellovibrio* to grow in the periplasm? We do not rule out that there may be other enzymes acting later in predatory development that allow the bdelloplast to expand to accommodate the progeny *Bdellovibrio*. It is also possible that solely the physical pressure of the internal *Bdellovibrio* cells pressing upon the stretchable peptidoglycan allows growth (the outward turgor pressure would be favourable for this, unlike the prey invasion process itself which fights against prey cell turgor pressure).

Predatorily specialised *bd3459 dacB*-like genes are retained in addition to the single housekeeping *bd3244*-like (Figure 5) gene in the genomes of other Bdellovibrios; the marine *Bdellovibrio*-like organism *Bacteriovorax marinus* [37] has a single predatory homologue (Bms2102) and housekeeping homologue (Bms2612) (Stine *et al.* 2010, unpublished data; GenBank ID: FQ312005.1). The retention of these genes emphasises the importance of their encoded products for fitness. The retention of duplicate predation-related genes is seen in other important *Bdellovibrio* systems like those encoding flagella [38] and their motors [39]. We speculate that *Bdellovibrio* can therefore repair gene damage by internal recombination from a “spare” gene sequence – clearly Bd3459 is more phenotypically active for prey cell wall rounding than Bd0816, and Bd0816 more active for tailgating prevention than Bd3459 so they do not directly substitute for each other totally. Bms2102 and Bd0816 are likely to share the same novel predatory structural adaptations, as observed when comparing the structure of Bd3459 to “regular” PBP4 proteins.

The presumed housekeeping Bd3244 protein has a more conventional PBP4 sequence and includes a predicted classical domain III (Figure 5). Notably it does not have a Sec sequence like those seen on the other Bd0816 and Bd3459 proteins. *Bdellovibrio* has Gram-negative typical A1γ peptidoglycan composition [40], but it may have modifications that render it immune/protected from attack, such as a particularly low number of cross-linked trimers/tetramers that Bd0816 and Bd3459 may cleave, or an immunity protein that acts to protect against self peptidoglycan-modification. Recently a type VI secretion system has been shown to be used by *Pseudomonas aeruginosa* to inject peptidoglycan hydrolases into the periplasm of Gram-negative bacteria [41]. *Pseudomonas* protects itself from self-intoxification with a periplasmic immunity protein. It remains to be determined how Bd0816/3459 are secreted into the prey periplasm and whether this involves novel transport mechanisms or solely Sec machinery. Unfortunately there is little available microscopic and electron-tomographic data on the nature of the junctions between *Bdellovibrio* and prey cells upon invasion, nor whether they involve direct membrane fusion junctions between the two outer membranes for example. So at present it is difficult to predict secretion mechanisms, as we cannot yet be sure if Bd0816 and Bd3459 proteins need to be secreted across the *Bdellovibrio* outer membrane, while it is invading the periplasm of prey, or whether the *Bdellovibrio* outer membrane is temporarily partially disrupted, giving direct access from one periplasm to the other. Further extensive tomographic studies are required inside bdelloplasts to answer this. We are working to determine whether or not *Bdellovibrio* peptidoglycan is susceptible to these Bd0816/3459 enzymes or is protected by an immunity protein(s) or by peptidoglycan modification. We are investigating the reason, but can confirm Thomashow and Rittenberg's hypothesis of 1978 that cell wall peptidases shape the prey cells into rounded bdelloplasts and to identify the structure, expression and function of those enzymes.

This work has characterised one pair of predatorily-adapted, PBP4-like enzymes from the arsenal of bacterially degrading and modifying proteins that *Bdellovibrio* encodes from its “predatosome” (genes transcribed in response to prey interaction) [14], and shown how their structure has evolved to rapidly modify the cell wall chemistries of diverse bacteria that *Bdellovibrio* prey upon to allow rapid, and preferentially singular access to a novel intracellular niche.

## Materials and Methods

### Strains and growth conditions

Bacterial strains and plasmids used during this study are listed in Table S1 in Text S1. Predatory *B. bacteriovorus* strains were routinely grown in Ca/HEPES buffer with *E. coli* as prey as previously described [38], typically yielding 2.5×10^8^ PFU ml^−1^. *E. coli* and *A. baumannii* were grown in YT broth at 37°C with 200 rpm shaking for 16 hours prior to use and typically yielded 1×10^9^ CFU ml^−1^.

### RNA isolation from predatory cycle and RT-PCR analysis

Synchronous predatory cultures of *B. bacteriovorus* HD100 and *E. coli* S17-1 were set up as previously described [38] and samples collected throughout the *Bdellovibrio* lifecycle as previously described [35] which were frozen. RNA was isolated from the frozen samples using the Promega SV total RNA isolation kit, the RNA quality verified, and the RT-PCR reactions carried out using the QIAGEN One-step RT-PCR kit all as described previously [35], [38]. RT-PCR conditions were as follows: one cycle of 50°C for 30 min, 95°C for 15 min, then 30 cycles (for *bd3244*) or 25 cycles (for *bd0816* and *bd3459*) of 94°C for 1 min, 48°C for 1 min, 72°C for 2 min, with a final extension of 72°C for 10 min before a final 4°C hold. Primers are detailed in Table S2 in Text S1. The RT-PCRs were performed on each of three independent sets of RNA from *B. bacteriovorus* HD100 time courses which all gave identical expression profiles.

### Gene deletion in *Bdellovibrio*

Chromosomal silent deletions of the *B. bacteriovorus* HD100 *bd0816* and *bd3459* reading frames and also the *bd0816* in the Δ*bd3459* mutant background were created using a modification of described methods [42]. Construction of each strain is described in Text S1.

### Electron microscopy and preparation of synchronous mini-infections

Ten ml of attack-phase *B. bacteriovorus* cells were sub-cultured every 24 hours for 3 days using *E. coli* S17-1 as prey using standard techniques [38]. After the third sub-culturing, 10 ml of the cleared prey lysate was filtered through a 0.45 µm pore-size filter to remove residual prey whilst allowing the *Bdellovibrio* to pass through. The filtrate was then centrifuged at 5,525× *g* for 20 minutes at 29°C and the cell pellet resuspended in 500 µl Ca/HEPES buffer. One ml of *E. coli* S17-1 prey was centrifuged at 17,000× *g* for 5 minutes at 29°C and resuspended in 5 ml Ca/HEPES buffer. The *Bdellovibrio* and *E. coli* cells were incubated separately at 29°C with 200 rpm shaking for 1 hour to recover. Aliquots of 500 µl of each of the *Bdellovibrio* and *E. coli* were mixed to start the invasion and were incubated at 29°C with 200 rpm shaking for 90 minutes. Grids were prepared using 2 µl of invaded culture stained with 1% phosphotungstic acid (pH 7.0) for 1 minute and observed at 80 kV using a JEOL 1200EX transmission electron microscope. Photographs were recorded onto photographic-negative plates, developed in house and scanned into a computer. Four independent experiments have looked at *Bdellovibrio* invasions by EM at various time points; the images in Figure 2 were all from the same experimental repeat, and the graph (Figure 2E) only used data from bdelloplasts at the 90 minutes post-infection time point. In order to measure the roundness coefficient, images of single bdelloplasts were traced using ImageJ software [43]; then an eclipse was automatically fitted to the outline. Using the ‘roundness’ function within the ImageJ software, the average bdelloplast roundness was calculated and statistical analyses performed.

### Measuring the time taken and tailgating frequency for *Bdellovibrio* to invade into the prey periplasm using time-lapse microscopy

Infections of *B. bacteriovorus* HD100, HD100 Δ*bd0816*, HD100 *Δbd3459* and HD100 Δ*bd0816* Δ*bd3459* preying upon *E. coli* K-12 MG1655 (chosen as it has a more uniformly identical population than S17-1) were set up by first sub-culturing *Bdellovibrio* to clear 3×10 ml cultures of prey and purifying them by filtration as described previously. Then 1 ml of *Bdellovibrio* lysate and 1 ml prey culture were pelleted by centrifugation at 17,000× *g* for 5 minutes at 29°C. The *Bdellovibrio* were resuspended in 50 µl of the residual Ca/HEPES and the prey was resuspended in 100 µl Ca/HEPES. Infection was started by mixing 50 µl each of *Bdellovibrio* and prey and incubated for 10 minutes at room temperature. Ten µl of the infection culture was immobilised on a glass microscope slide covered in a 1% agarose-Ca/HEPES pad and visualised using a 100× objective on a Nikon Eclipse E600 microscope equipped with a Prior Scientific H101A XYZ motorised stage to allow precise revisiting of 6 fields of view at 1 min intervals. Images were captured using Simple PCI software and processed using the “sharpen” then “smooth” tools (see [17] for further details).

Invasion times were measured by counting the number of frames (1 frame = 1 min) taken from the first sign of invasion (i.e. lateral movement of *Bdellovibrio* cell body into the prey cell wall) to being completely inside the bdelloplast. Only prey cells with a single attached *Bdellovibrio* at the start of the time-lapse sequence were measured. *Bdellovibrio* length (pole-pole) was measured using the Simple PCI software. The same videos were used to analyse the frequency of ‘tailgating’ *E. coli* entries by *Bdellovibrio*, but in this analysis, only prey cells with two attached *Bdellovibrio* were used (Figure 4). These were found by observation of time lapse movies. Both *Bdellovibrio* had to be attached for at least 10 minutes in order to constitute an ‘attempted invasion’; those which were attached for less than 10 minutes were treated as being randomly attached and not attempting invasion, so “attached” in the subsequent explanation refers to those ≥10 min-attached cells. Tally counts were made in the observed prey-invasion categories as follows: a) ‘single’, where two *Bdellovibrio* are attached but only one enters; b) ‘synchronous’, where two *Bdellovibrio* are attached and both invade at the same time; or c) ‘tailgating’, where two *Bdellovibrio* are attached, one invades and the second invades after the first invasion is complete. In this case, the elapsed time before the second invasion was noted. In all cases, data were taken from at least two independent experimental replicates, with at least 20 data points in each, from at least five fields of view. (Although we had noted the relative proximity of the two invading *Bdellovibrio*, we did not find that this strongly influenced the outcome of tailgating).

### Heterologous overexpression of an active and inactive form of *Bdellovibrio* Bd3459 in *E. coli*

The construction of the heterologous overexpression vectors is described in detail in Text S1. Essentially, a modified version of the pBADHisA (Invitrogen) was used (the native His tag was removed and a kanamycin resistance cassette introduced) and the *B. bacteriovorus* HD100 *bd3459* reading frame was cloned in-frame under the control of the *araBAD* promoter. As a control, a version with the active site serine mutagenised to alanine (S70A) was also constructed (see Text S1). Both constructs were transformed into *E. coli* TOP10.

Heterologous overexpression experiments were performed by immobilising overnight (16 hr) *E. coli* TOP10 cultures containing the plasmid constructs onto a 1% agarose-YT pad containing kanamycin^50^ and 0.2% (v/v) L-arabinose (to induce expression at time zero) and these were subjected to time-lapse microscopy (2.5 minute resolution) as described previously. At least two independent experimental replicates were performed in all cases.

### Cloning of overexpression construct for Bd3459 structure determination, protein expression and purification

The overexpression construct for Bd3459 structure determination was created as described in detail in Text S1. Briefly, a restriction free process [44] was used to clone the region encoding the secreted form of Bd3459 into a modified version of the expression plasmid pET41 (Novagen, altered to remove GST) and transformed into the *E. coli* expression strain BL21 (DE3). For protein expression, cells were grown at 37°C until reaching an OD_600_ of ≈0.8, then gene expression induced with 1 mM IPTG for 20 hours at 20°C. Harvested cells (≈10 g from 1.5 L cell culture in 2× YT media) were resuspended by tumbling in 30 ml buffer A (20 mM HEPES pH 7.2, 0.3 M NaCl, 20 mM imidazole, 5% v/v glycerol and 0.1% w/v Tween20) and lysed using sonication. Unbroken cells were pelleted by centrifugation at 6000× g for 20 minutes, the supernatant clarified by a second centrifugation at 200,000× g for 1.5 hours, and the final supernatant applied to a 1 ml Hi-Trap His column, pre-equilibrated in buffer A. Fractions were eluted in a stepwise manner, using buffer A containing 40 and 300 mM imidazole. Approximately pure fractions of Bd3459 were dialyzed overnight in buffer B (10 mM Tris pH 7.2, 200 mM NaCl) and concentrated to a protein concentration of ≈35 mg ml^−1^.

### Crystallization and cryoprotection

Crystals were grown by the hanging drop method at 18°C, using 1 µl of protein solution mixed with an equal volume of reservoir solution. Initial crystallization conditions were identified in 0.1 M MES pH 6.5, 0.2 M ammonium sulphate, 30% w/v PEG 5000 MME, which resulted in thin, unusable needles. Addition of 16% v/v glycerol to the reservoir solution improved sample size and shape, leading to the growth of diffraction-quality crystals. Cryoprotection was attained by sequential addition of increments of mother liquor supplemented with 20% (v/v) ethylene glycol, followed by subsequent flash cooling in liquid nitrogen. Derivitized crystals for phasing purposes were obtained by soaking native crystals in cryoprotection solution containing 10 mM K_2_PtCl_4_ for 30 minutes before flash cooling.

### Data collection and structure determination

Diffraction data were collected at beamline ID23-1 of the ESRF, Grenoble (native data) and at beamline I03 of the Diamond Light Source, Oxford (platinum derivative). Data were processed using XDS [45] and SCALA, and data file manipulations performed using the CCP4 suite of programs [46]. Molecular replacement attempts with existing transpeptidase/carboxypeptidase structures resulted in poor quality maps, due to poor structural agreement and no possibility of averaging (a single copy of Bd3459 is present in the asymmetric unit). Excellent SIRAS phases for the Pt derivative data were obtained using PHENIX autosolve [47], which located 6 sites at 2.9 Å resolution (4 of which were via reaction with surface-exposed methionine sidechains). The overall figure of merit (FOM) for phasing was 0.38. Density modification yielded a readily interpretable map (aided by a relatively high solvent content of 69% and phase extension to 1.45 Å), and the molecule was built manually using COOT [48]. Model refinement used PHENIX [47], refining individual B-factors with additional TLS parameters. The final model is of excellent stereochemical quality (refer to Table 1), with 91.4 and 8.3% of residues in the most-favoured and additionally favoured regions of the Ramachandran plot respectively; a single residue (A336) is the solitary outlier, but is present in a tight turn modelled into excellent, unambiguous electron density.

**Table 1:** Data collection and refinement statistics.

	Native	Pt Derivative
**Data collection**		
Space group	P3_1_21	P3_1_21
Cell dimensions		
*a*, *b*, *c* (Å)	125.2, 125.2, 81.4	124.8, 124.8, 81.6
α β γ (°)	90, 90, 120	90, 90, 120
Resolution (Å)	1.45 (1.53–1.45)*	2.9 (3.06–2.9)
*R*_sym_	6.8 (46.6)	9.4 (19.2)
*R*_pim_	4.7 (36.3)	2.9 (6.0)
*I*/σ*I*	11.7 (1.7)	28.6 (14.1)
Completeness (%)	98.6 (92.0)	99.8 (100)
Redundancy	4.4 (2.7)	21.2 (21.7)
**Refinement**		
Resolution (Å)	1.45	-
No. reflections	127960	-
*R*_work_/*R*_free_	16.6/18.2	-
No. of atoms/average B factor		
Protein	3206/27.8	
Ligand	33/41.1	
Water	441/31.5	
R.m.s. deviations		
Bond lengths (Å)	0.022	-
Bond angles (°)	1.9	-

*Values in parentheses are for highest-resolution shell.

### Enzyme activity of Bd3459 and Bd3459 (S70A)

Twenty µg of Bd3459 or the active-site mutant protein Bd3459 (S70A) were incubated in 200 µl of 20 mM sodium phosphate, pH 4.8 for 3 h at 37°C with 0.25 mg ml^−1^ peptidoglycan from *E. coli* CS703-1, which is enriched in pentapeptide-containing muropeptides [49]. Control samples contained either no protein or Bd3459 that had been pre-incubated with 0.4 mg ml^−1^ ampicillin for 30 min at 37°C. The reaction was stopped by boiling for 10 min. Muropeptides were generated by incubation with 40 µg ml^−1^ cellosyl (provided by Höchst AG, Frankfurt, Germany) for 18 h at 37°C. Samples were boiled for 10 min and centrifuged for 15 min at 16,000× g. A 150 µl aliquot of the supernatant was reduced with sodium borohydride and analysed by HPLC as published [50] except that a Prontosil 120-3-C18 AQ reversed-phase column (250×4.6 mm, 3 µm) from Bischoff (Leonberg, Germany) was used. The structures of major muropeptides shown in Figure 7B were assigned based on the retention time in HPLC (Figure 7A) when comparing to well-characterized *E. coli* muropeptide standards the structures of which have previously been determined by mass spectrometry and chemical composition [50].

### Bocillin-FL binding assay

Bd3459 or Bd3459 (S70A) (75 µg ml^−1^) were incubated in 40 µl of 20 mM sodium phosphate, pH 4.8 with 1.25 µg ml^−1^ Bocillin-FL for 10 min at 37°C. Control samples were pre-incubated with 2.5 µg ml^−1^ ampicillin prior to Bocillin-FL-labelling. The reaction was stopped by adding 20 µl of SDS-PAGE loading buffer and boiling for 10 min. A 10 µl aliquot of each sample was analysed by 12% SDS-polyacrylamide gel electrophoresis. The Bocillin-FL fluorescence emission signal at 520 nm was scanned with a Typhoon reader (GE Healthcare, Little Chalfont, UK) upon excitation at 488 nm. Proteins were subsequently stained with Coomassie Blue.

## Supporting Information

Figure S1 Mixture of light and electron microscopy images showing larger fields of view depicting the morphologies of multiple bdelloplasts for each invading strain of *B. bacteriovorus*. A = wild type HD100; B = HD100 ΔBd0816; C = HD100 ΔBd3459; D = HD100 ΔBd0816 ΔBd3459.
(TIF)

Figure S2 Frequency distribution for duration of *Bdellovibrio* attachment (first contact until invasion) on *E. coli* prey.
(PNG)

Figure S3 Frequency distribution for duration of *Bdellovibrio* attachment (first contact until invasion) on *A. baumannii* prey.
(PNG)

Figure S4 Frequency distribution for duration of *Bdellovibrio* invasion (first movement into prey until completely within) on *E. coli* prey.
(PNG)

Figure S5 Frequency distribution for duration of *Bdellovibrio* invasion (first movement into prey until completely within) on *A. baumannii* prey.
(PNG)

Figure S6 Frequency distribution for overall duration (*Bdellovibrio* attachment+invasion) on *E. coli* prey.
(PNG)

Figure S7 Frequency distribution for overall duration (*Bdellovibrio* attachment+invasion) on *A. baumannii* prey.
(PNG)

Figure S8 Periplasmic preparations of *E. coli* Top10 cells containing expression construct producing Bd3459 WT and S70A protein grown in inducing (in) and uninducing (un) conditions (+/−0.2% arabinose) for 1 hour. Shows that the Bd3459 protein is present in the periplasm and is more abundant when induced. Pure Bd3459 protein (no signal sequence) was used as a positive control, and ran at the expected size of ∼46 kDa. Amounts of protein loaded was matched by Lowry Assay to 14 mg/ml. L = Benchmark ladder (5 µl).
(TIF)

Text S1 Detailed method of construction of vectors and mutant strains used in this study, incorporating Table S1 (list of bacterial strains and plasmids used in this study) and Table S2 (list of PCR primers used in this study), as well as references for the above.
(DOC)

Video S1 Time lapse movie showing wild type *B. bacteriovorus* HD100 invading an *E. coli* K-12 MG1655 cell. In the centre of the movie, an attached *Bdellovibrio* enters the prey, and the ‘rounding up’ of the cell into a bdelloplast begins just before invasion, and finishes concurrently with the end of invasion. Video is at 7 frames per second.
(MP4)

Video S2 Time lapse movie showing wild type *B. bacteriovorus* HD100 invading an *A. baumannii* cell. On the left hand side of the movie, an attached *Bdellovibrio* enters the prey, and the ‘rounding up’ of the cell into a bdelloplast begins slightly earlier before invasion compared to invasion of *E. coli*. In addition, the rounding is completed prior to the end of invasion. Video is at 7 frames per second.
(MP4)

Video S3 Time lapse movie showing a double gene deletion mutant of *B. bacteriovorus* HD100 (Δ*bd0816Δbd3459*) invading an *E. coli* K-12 MG1655 cell. An attached *Bdellovibrio* enters the prey, without the characteristic ‘rounding up’ of the cell. Video is at 7 frames per second.
(MP4)

Video S4 Time lapse movie showing a double gene deletion mutant of *B. bacteriovorus* HD100 (Δ*bd0816Δbd3459*) invading an *A. baumannii* cell. On the right hand side of the movie, an attached *Bdellovibrio* enters the prey, without the characteristic ‘rounding up’ of the cell. Video is at 7 frames per second.
(MP4)

Video S5 Time lapse movie showing a single wild type *B. bacteriovorus* HD100 cell invading an *A. baumannii* cell on the right hand side, which, concurrently with invasion becomes deformed on the left hand side of the cell due to being in contact with another *A. baumannii* cell. Video is at 7 frames per second.
(MP4)

Video S6 Time lapse movie showing wild type *B. bacteriovorus* HD100 cells invading two touching *E. coli* cells in the centre of the field of view. Although they both round up into a bdelloplast, neither bdelloplast deforms when in contact with the other. Video is at 7 frames per second.
(MP4)

Video S7 Time lapse movie showing two wild type *B. bacteriovorus* HD100 cells both trying to invade one *E. coli* K-12 MG1655 cell, with only one successful. One of the *Bdellovibrio* successfully invades the prey cell whereas the other is attached for long enough to constitute an attempted invasion but never does. Video is at 7 frames per second.
(MP4)

Video S8 Time lapse movie showing two wild type *B. bacteriovorus* HD100 cells both trying to invade one *E. coli* K-12 MG1655 cell, with both successfully invading synchronously. The top left hand side of the movie shows two *Bdellovibrio* attached to a prey cell, and both invade at opposite poles of the prey cell at the same moment. Video is at 7 frames per second.
(MP4)

Video S9 Time lapse movie showing two wild type *B. bacteriovorus* HD100 cells both trying to invade one *E. coli* K-12 MG1655 cell, with both successfully invading in a ‘tailgating’ manner. One of the attached *Bdellovibrio* fully enters the prey cell and just as it completes invasion, the other *Bdellovibrio* starts and successfully invades too. Video is at 7 frames per second.
(MP4)

Video S10 Time lapse movie showing two cells of a double gene deletion mutant of *B. bacteriovorus* HD100 (Δ*bd0816Δbd3459*) invading one *E. coli* K-12 MG1655 cell, with only one successful. Video is at 7 frames per second.
(MP4)

Video S11 Time lapse movie showing two cells of a double gene deletion mutant of *B. bacteriovorus* HD100 (Δ*bd0816Δbd3459*) invading one *E. coli* K-12 MG1655 cell, with both successfully invading synchronously. Video is at 7 frames per second.
(MP4)

Video S12 Time lapse movie showing two cells of a double gene deletion mutant of *B. bacteriovorus* HD100 (Δ*bd0816Δbd3459*) invading one *E. coli* K-12 MG1655 cell, with both successfully invading in a ‘tailgating’ manner. The left hand side of the movie shows a prey cell with two attached *Bdellovibrio*, in which one fully enters the prey before there is a delay and then the other *Bdellovibrio* successfully invades. Video is at 7 frames per second.
(MP4)

Video S13 Time lapse movie showing *E. coli* TOP10 cells expressing wild type Bd3459 protein on an YT-agarose pad from 0–6 hours after inducing protein expression. The movie shows several cells that initially begin to elongate, but after 1.5 hours have started to show abnormal morphologies. Over the next few hours, the cells become increasingly large and show bleb-like projections, due to the lack of cell wall integrity being able to resist the hypotonic medium, and eventually the cells lyse. Video is at 3 frames per second.
(MP4)

Video S14 Time lapse movie showing *E. coli* TOP10 cells expressing inactive (S70A) Bd3459 protein on an YT-agarose pad from 0–6 hours after inducing protein expression. The movie shows several cells that after a short lag phase begin replicating exponentially as normal *E. coli* do. Video is at 3 frames per second.
(MP4)

Video S15 Time lapse movie showing *E. coli* TOP10 cells expressing wild type Bd3459 protein on a M-medium-agarose pad from 0–6 hours after inducing protein expression. The movie shows several cells that initially being to elongate, but after approximately 2 hours have neatly rounded up (reminiscent of bdelloplasts) as they are osmotically supported by the hypertonic medium. Eventually the cells become larger and more irregular, but this could have been due to the tonicity of the media changing as the agarose pad dried out. Video is at 3 frames per second.
(MP4)

Video S16 Time lapse movie showing *E. coli* TOP10 cells expressing inactive (S70A) Bd3459 protein on a M-medium-agarose pad from 0–6 hours after inducing protein expression. The movie shows several cells that after an extended lag phase begin replicating slowly but exponentially as normal *E. coli* do. Video is at 3 frames per second.
(MP4)
