# Supplementary figures and images for: Specialized Peptidoglycan Hydrolases Sculpt the Intra-bacterial Niche of Predatory Bdellovibrio and Increase Population Fitness
Source: PLoS Pathog. 2012 Feb 9;8(2):e1002524. doi: 10.1371/journal.ppat.1002524 (PMC3276566; doi:10.1371/journal.ppat.1002524)

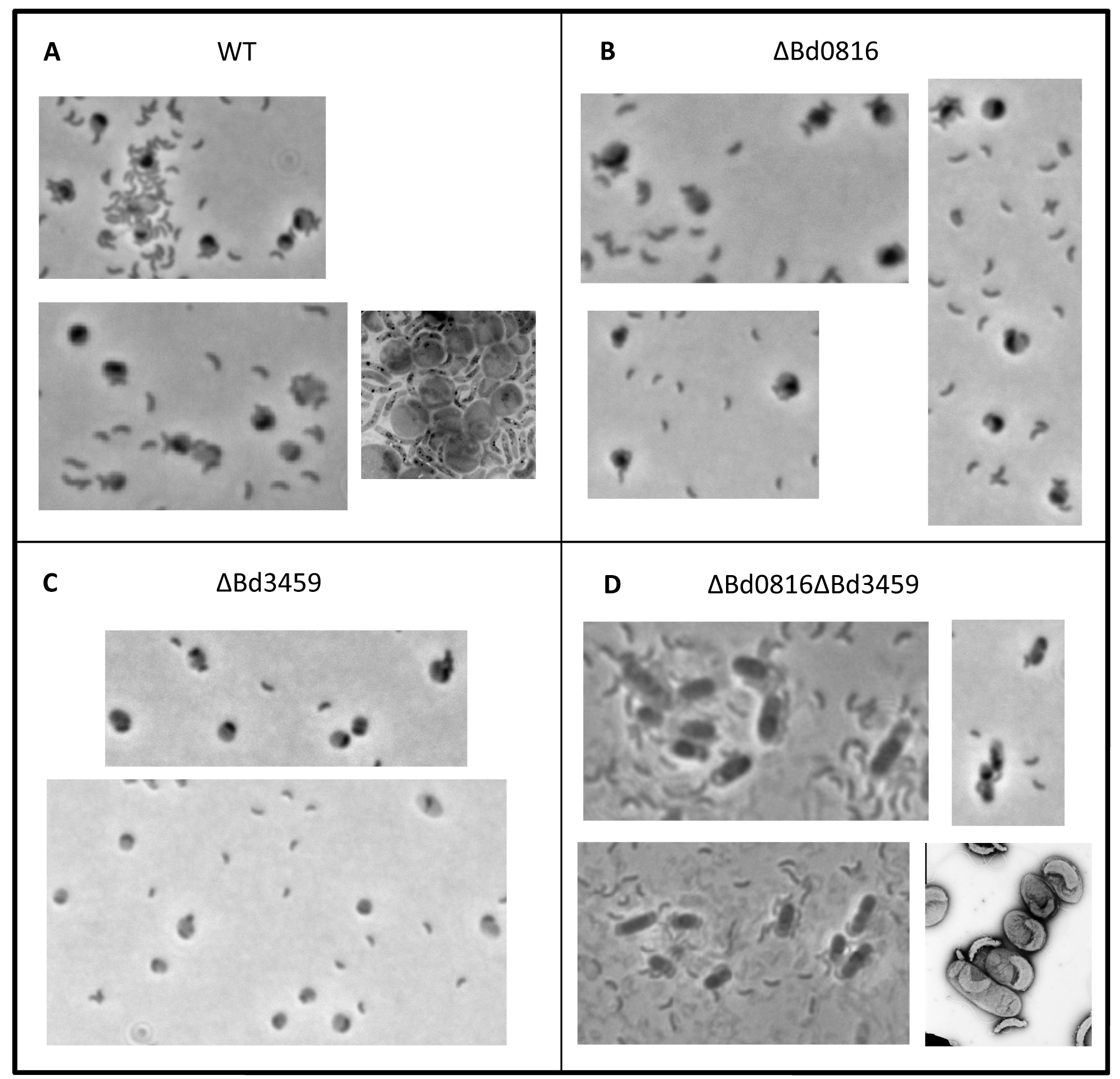

Supplement: Figure S1 — Mixture of light and electron microscopy images showing larger fields of view depicting the morphologies of multiple bdelloplasts for each invading strain of B. bacteriovorus. A = wild type HD100; B = HD100 ΔBd0816; C = HD100 ΔBd3459; D = HD100 ΔBd0816 ΔBd3459. (TIF) [file ppat.1002524.s001.tif]

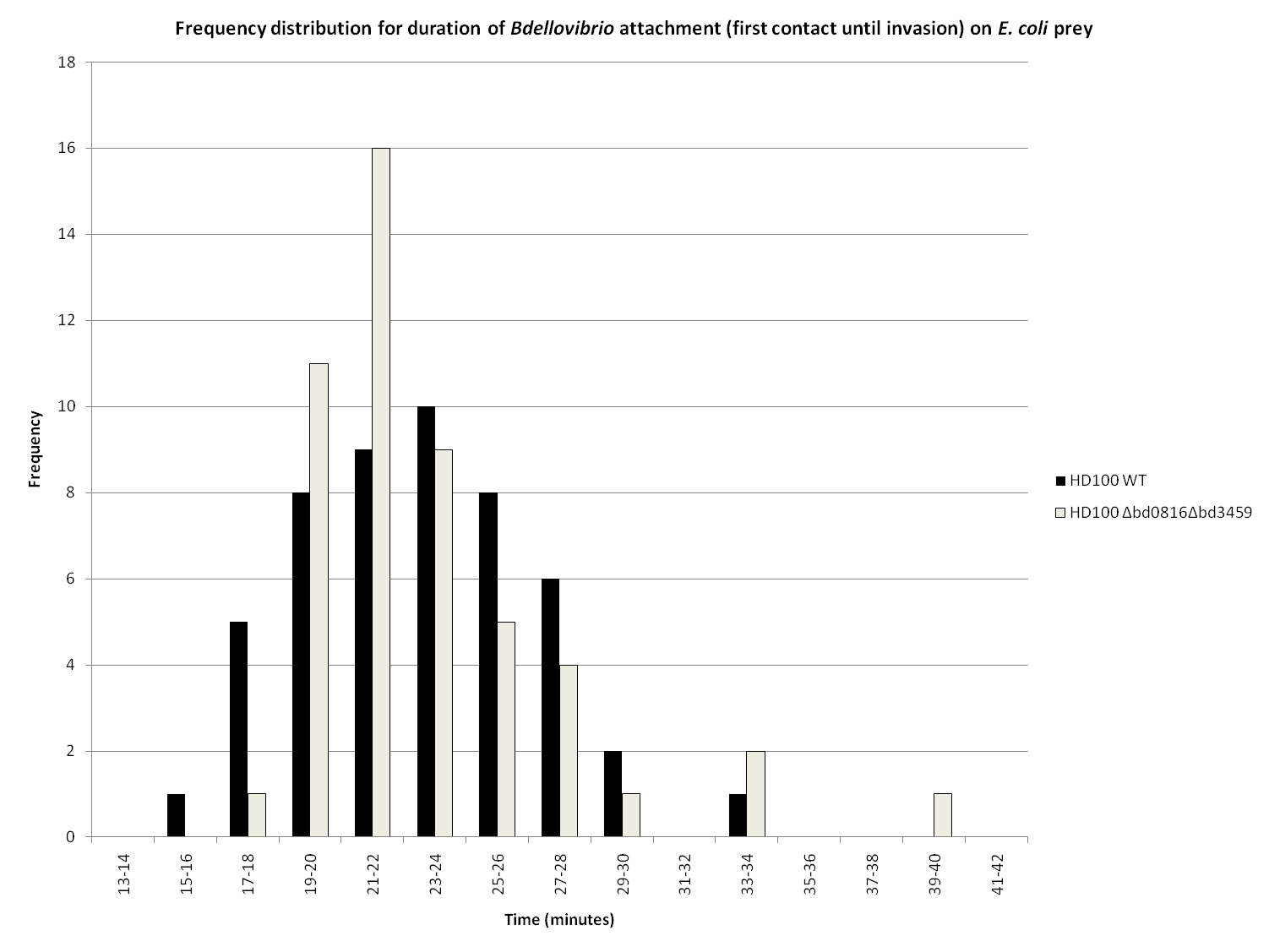

Supplement: Figure S2 — Frequency distribution for duration of Bdellovibrio attachment (first contact until invasion) on E. coli prey. (PNG) [file ppat.1002524.s002.png]

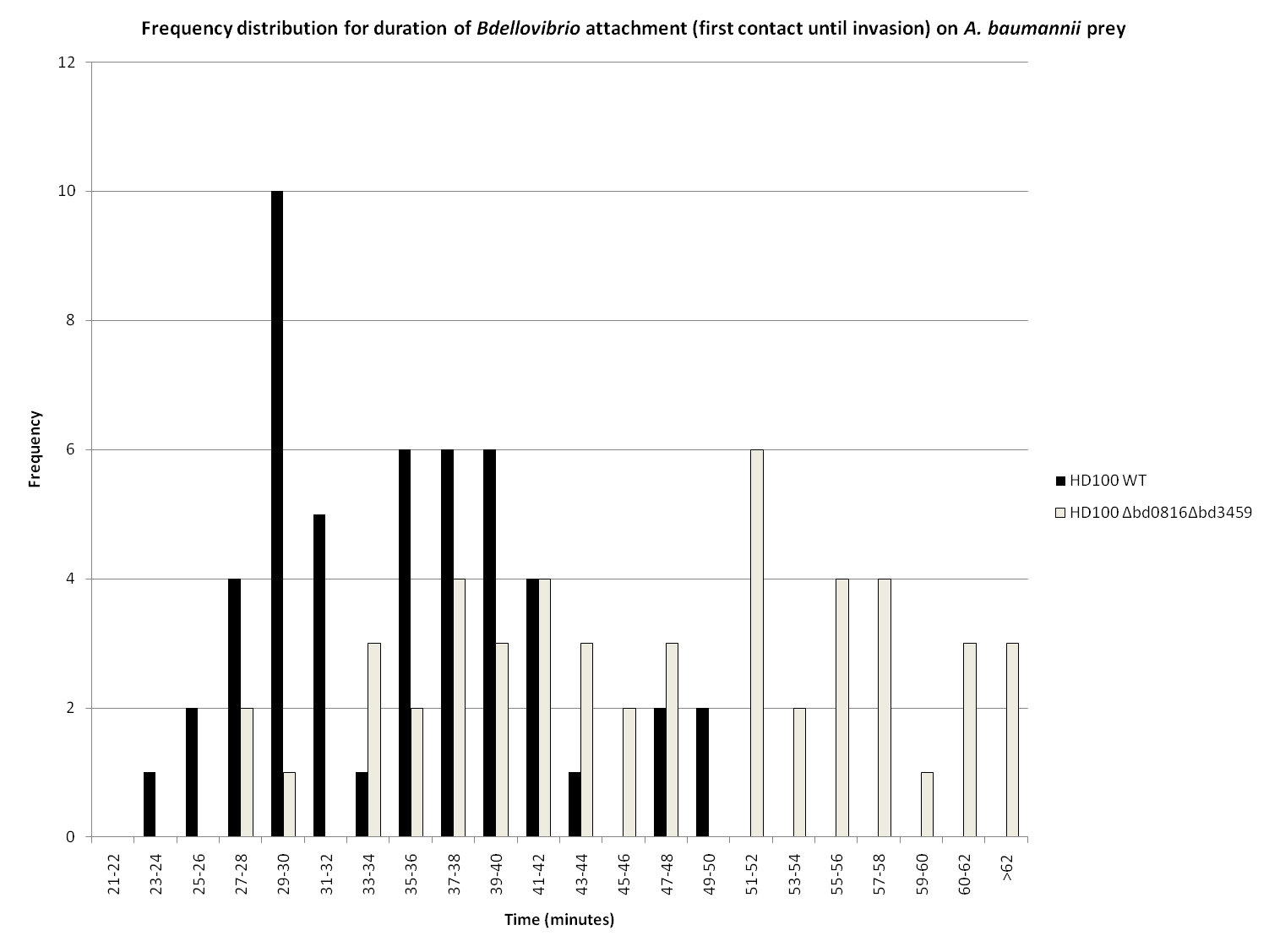

Supplement: Figure S3 — Frequency distribution for duration of Bdellovibrio attachment (first contact until invasion) on A. baumannii prey. (PNG) [file ppat.1002524.s003.png]

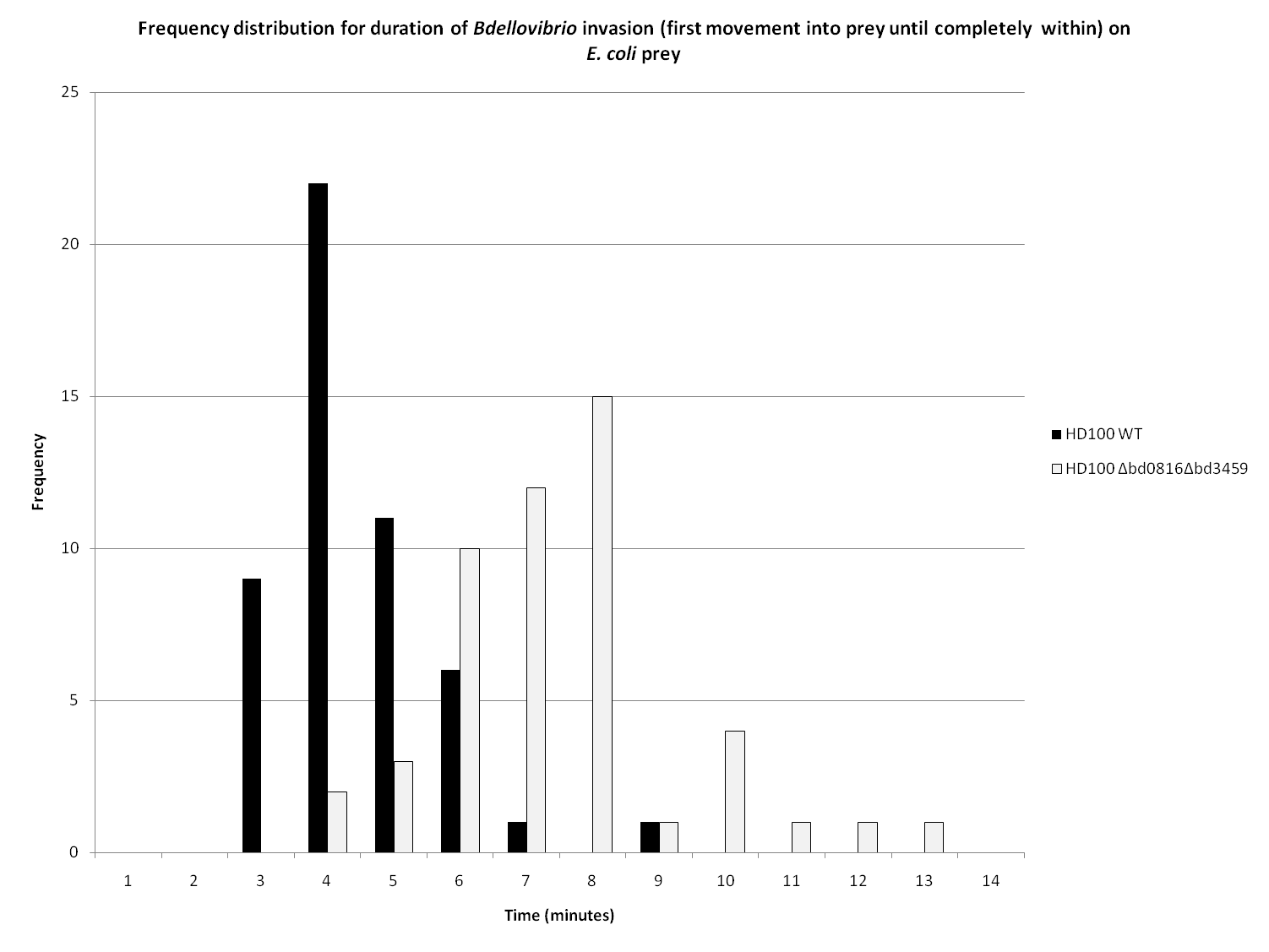

Supplement: Figure S4 — Frequency distribution for duration of Bdellovibrio invasion (first movement into prey until completely within) on E. coli prey. (PNG) [file ppat.1002524.s004.png]

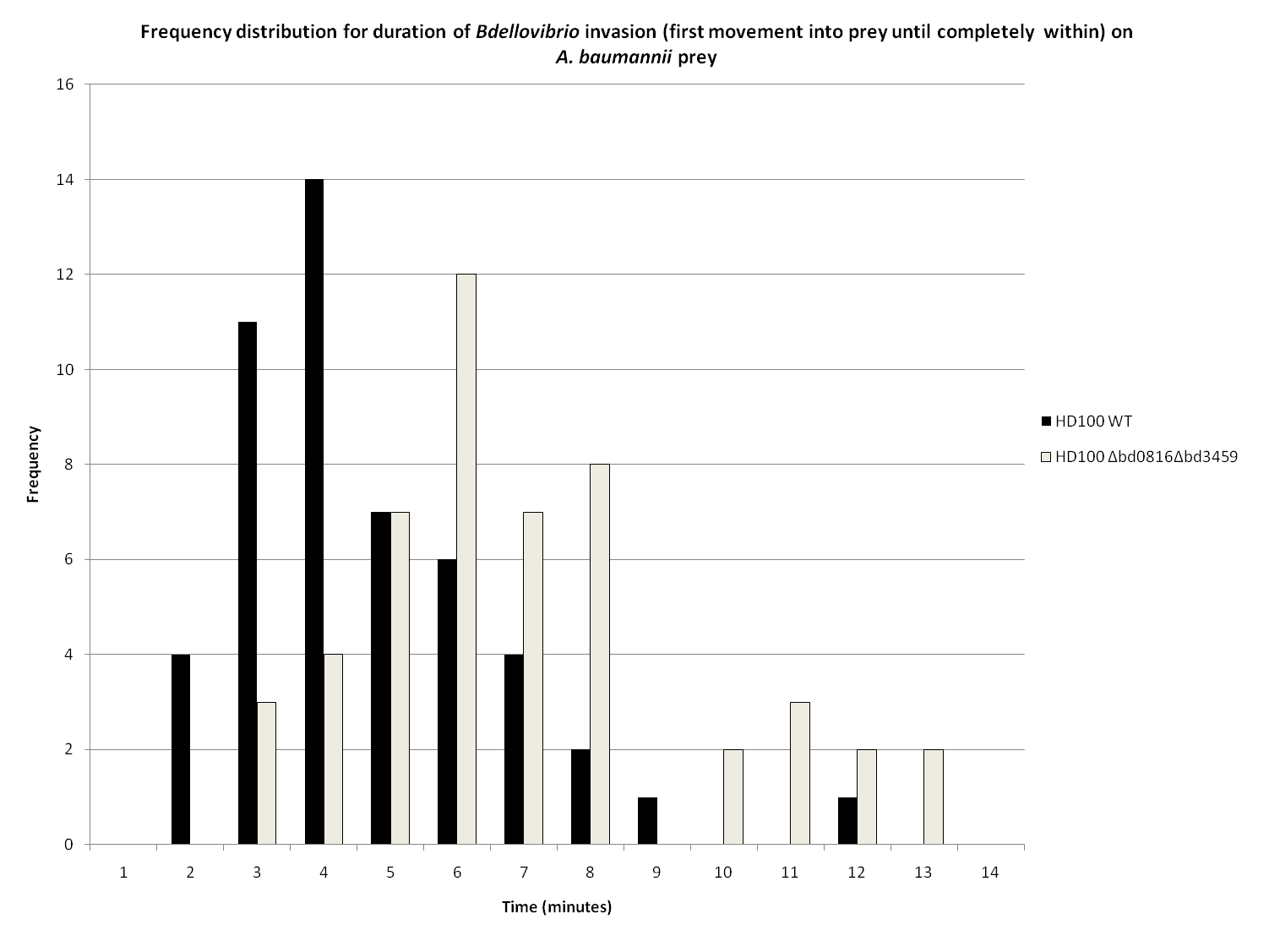

Supplement: Figure S5 — Frequency distribution for duration of Bdellovibrio invasion (first movement into prey until completely within) on A. baumannii prey. (PNG) [file ppat.1002524.s005.png]

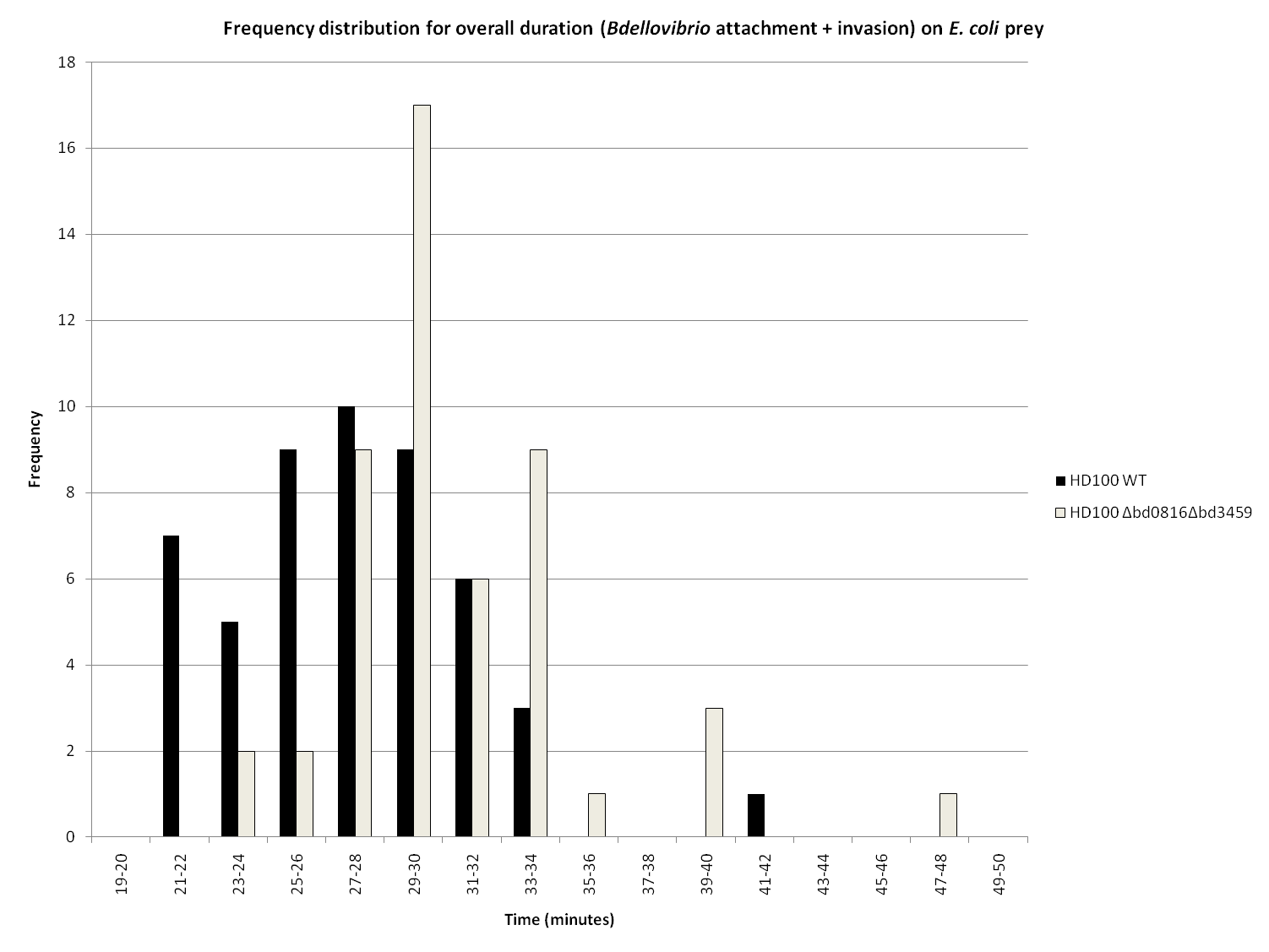

Supplement: Figure S6 — Frequency distribution for overall duration (Bdellovibrio attachment+invasion) on E. coli prey. (PNG) [file ppat.1002524.s006.png]

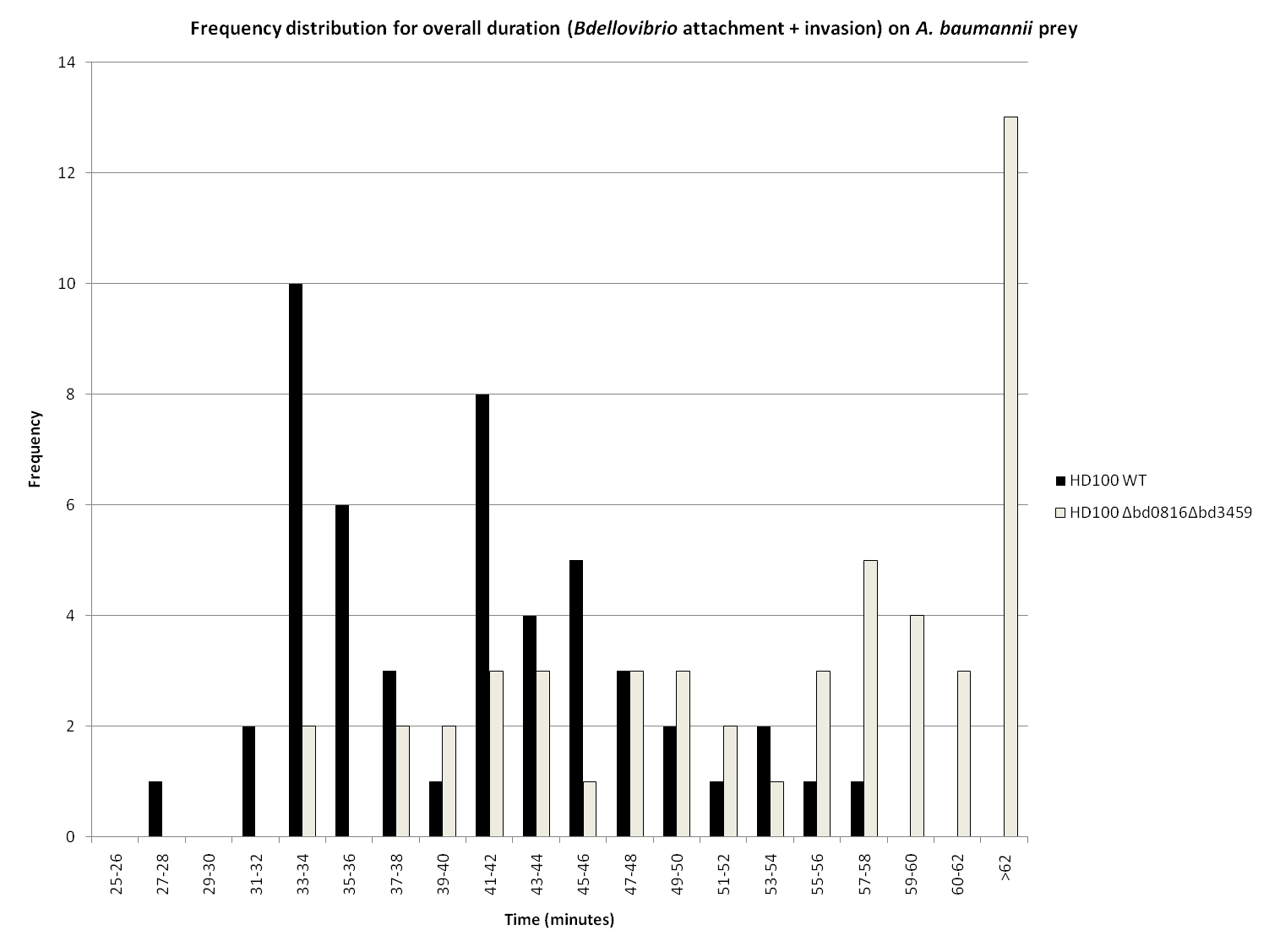

Supplement: Figure S7 — Frequency distribution for overall duration (Bdellovibrio attachment+invasion) on A. baumannii prey. (PNG) [file ppat.1002524.s007.png]

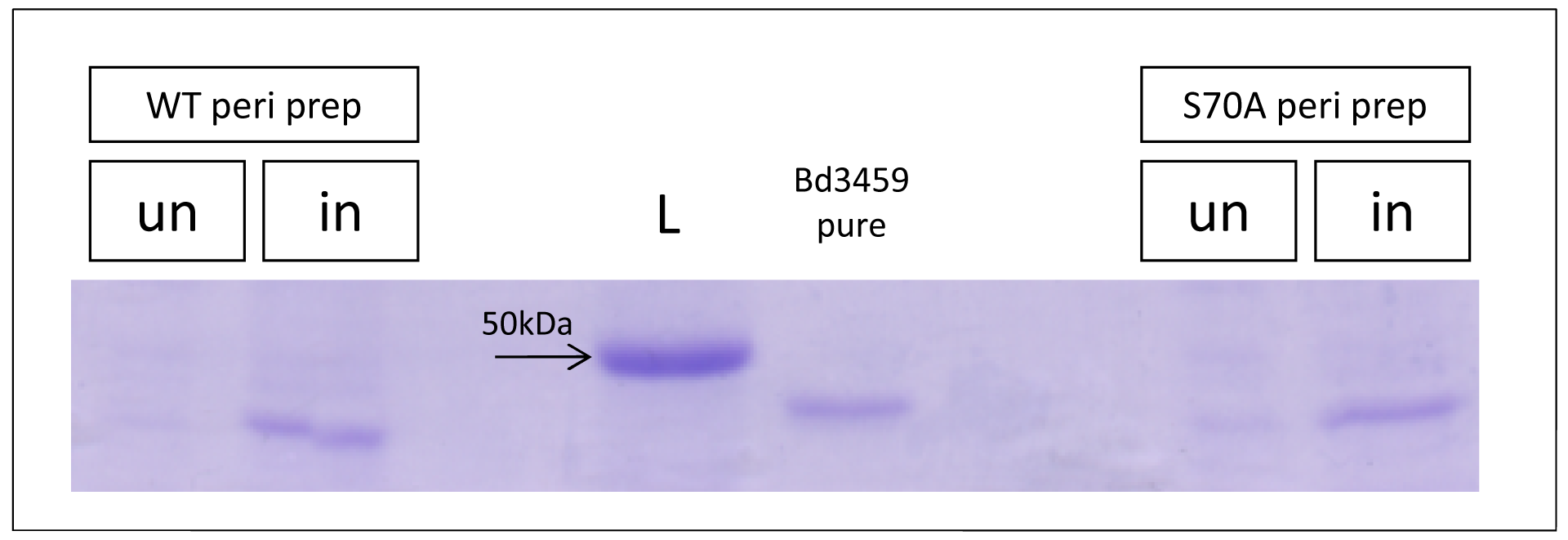

Supplement: Figure S8 — Periplasmic preparations of E. coli Top10 cells containing expression construct producing Bd3459 WT and S70A protein grown in inducing (in) and uninducing (un) conditions (+/−0.2% arabinose) for 1 hour. Shows that the Bd3459 protein is present in the periplasm and is more abundant when induced. Pure Bd3459 protein (no signal sequence) was used as a positive control, and ran at the expected size of ∼46 kDa. Amounts of protein loaded was matched by Lowry Assay to 14 mg/ml. L = Benchmark ladder (5 µl). (TIF) [file ppat.1002524.s008.tif]
