# Supplementary material for: Specialized Peptidoglycan Hydrolases Sculpt the Intra-bacterial Niche of Predatory Bdellovibrio and Increase Population Fitness
Source: PLoS Pathog. 2012 Feb 9;8(2):e1002524. doi: 10.1371/journal.ppat.1002524 (PMC3276566; doi:10.1371/journal.ppat.1002524)
Supplement: Text S1 — Detailed method of construction of vectors and mutant strains used in this study, incorporating Table S1 (list of bacterial strains and plasmids used in this study) and Table S2 (list of PCR primers used in this study), as well as references for the above. (DOC) [file ppat.1002524.s009.doc]

**Text S1.** Method of construction of vectors and mutant strains used in this study.

**Construction of gene deletion mutants in *Bdellovibrio.*** Chromosomal gene deletions (silent knockouts) of *bd0816* and *bd3459* were performed using a modified method related to those of Roschanski and co-workers [1] and Steyert and co-workers [2]. Approximately 1 Kb of flanking DNA from upstream and downstream of the gene of interest was PCR amplified and fused together (using primers given in Table S2 in Text S1) such that the first two and last 4 amino acids of the otherwise deleted ORF remained, and a restriction site introduced after the stop codon. These approximately 2 Kb constructs were then cloned into the high copy number vector pUC19 (amp^r^), transformed into *E. coli* DH5α and confirmed, which allowed for a larger quantity of construct DNA to be accumulated than PCR alone. The construct DNA was then transferred into the low copy number suicide vector pK18*mobsacB* (kan^r^) which was transformed into *E. coli* DH5α, and fully confirmed by commercial sequencing (Source Bioscience). The confirmed plasmid was then transformed into the *E. coli* strain S17-1 [3] as a donor strain for the subsequent conjugation into *B. bacteriovorus* HD100 in the presence of kanamycin (50 µg ml^-1^) and plated onto double layer overlay plates on lawns of kanamycin resistant S17-1 (pZMR100) prey; this resulted in single plaques of recombinant merodiploid *Bdellovibrio* cells that contained the pK18*mobsacB* plasmid with the knockout construct in a single crossover using the homologous flanking DNA. These merodiploid *Bdellovibrio* cells were subjected to counter-selection with 5% sucrose during culturing in Ca/HEPES buffer and *E. coli* S17-1 prey and were again plated to single plaques (this time with no antibiotic selection). Resulting plaques were formed by *Bdellovibrio* which had been forced by the counter-selection to undergo a second crossover and therefore were either revertant to wild type or (rarely) contained the deletion construct in place of the wild type gene. The plaques were picked into 96 well plates containing Ca/HEPES and *E. coli* S17-1 prey which when cleared were PCR screened for the deletion using BioTaq DNA polymerase (Bioline) and 2 µl of the culture directly. Any potential knockouts were confirmed using Southern Blotting, sequencing and by RT-PCR using RNA derived from mutant strains to confirm the lack of a transcript of the deleted gene.

**Construction of heterologous overexpression vectors.** In order to heterologously overexpress the native *B. bacteriovorus* HD100 *bd3459* gene in *E. coli* it was necessary to use an extremely tightly controlled vector system as it became apparent that leakage from promoters was causing lysis of the cells and therefore Bd3459 was a toxic gene in *E. coli*. The pBADHisA vector system (Invitrogen) has the extremely tightly regulated *araBAD* promoter (L-arabinose inducible) which is further repressed by the addition of glucose and the repressor encoded by *araC*. However the vector also contains an N-terminal His-tag which needed to be removed and was done so by digesting the pBADHisA DNA with *NcoI* and *HindIII* restriction enzymes which removed the vector start codon and following His tag until the MCS. Primers (see Table S2 in Text S1) were designed to amplify the whole *bd3459* reading frame from the HD100 chromosome with an inserted *Nco*I site on the 5’ end (which restored the vector start codon and kept the *bd3459* ORF in frame), and an inserted *Hind*III site on the 3’ end. The active site serine to alanine (S70A) mutant was created by site directed PCR mutagenesis (see primers in Table S2). The codon encoding alanine which was most frequent in the *Bdellovibrio* genome (GCC) was chosen. Both clones (called pBAD::Bd3459 and pBAD::Bd3459(S70A)) were transformed into *E. coli* TOP10 (cannot metabolise L-arabinose) and fully confirmed. Because the pBAD vector series are based on ampicillin resistance, it was necessary to introduce a kanamycin resistance cassette (as ampicillin in a penicillin-analogue which irreversibly binds to penicillin binding proteins such as PBP4 and Bd3459). The pBAD::Bd3459 and S70A clones were blunt-cut in the centre of the *bla* gene with *Sca*I and a kanamycin resistance cassette from a pUC4K *Hinc*II fragment was cloned in using kanamycin selection (25 µg ml^-1^). These were again fully confirmed (and that the insert was in the same orientation) before being transformed *into* *E. coli* TOP10 in which the overexpression experiments were performed.

**Construction of overexpression vector for protein expression, purification and crystallization.** For protein purification, primers (see Table S2 in Text S1) were designed to amplify the secreted form of Bd3459 (starting at K39 with mutation of A38 to become the new N-terminal methionine and placing a LEHHHHHH tag on the C-terminal end of the protein) and also to have homology to the expression plasmid pET41 (Novagen, altered to remove GST) for construction in a restriction-free process [4]. The resulting PCR product was used in a second round of PCR with the modified pET41 vector as template, after which the template DNA was digested away by the DNA methylation-sensitive enzyme *Dpn*I leaving the nascent DNA containing the desired construct. This was transformed into *E. coli* DH5α and confirmed by sequencing before being introduced into the *E. coli* expression strain BL21 (DE3).

**Table S1.** Bacterial strains and plasmids.

| **Strain or Plasmid** | **Genotype or description** | **Reference** |
| --- | --- | --- |
| ***Escherichia coli*** | | |
| S17-1 | *thi,pro,hsdR*^-^,*hsdM*^+^,*rec*A; integrated plasmid RP4-Tc::Mu-Kn::Tn*7*; used as donor for conjugating plasmids into *Bdellovibrio* | [5] |
| DH5α | F′ *endA1* *hsdR17*(r_k_^–^m_k_^–^) *supE44 thi-1* *recA1 gyrA* (Nal^r^) *relA1*∆(*lacIZYA-argF*) U169 *deoR*(φ80d*lac*∆(*lacZ*)M15); used as a cloning host strain | [6] |
| S17-1::pZMR100 | S17-1 strain containing pZMR100 plasmid used to confer Km^r^; used as Km^r^ prey for *Bdellovibrio* | [5,7] |
| MG1655 | F- lambda- *ilvG*- *rfb*-50 *rph*-1 (K12 derivative) | [8] |
| BL21 (DE3) | F^–^ ompT gal dcm lon hsdS_B_(r_B_^-^ m_B_^-^) λ(DE3 [lacI lacUV5-T7 gene 1 ind1 sam7 nin5]) | Invitrogen |
| TOP10 | F- *mcr*A Δ(*mrr-hsd*RMS-*mcr*BC) φ80*lac*ZΔM15 Δ*lac*X74 *deo*R *rec*A1 *ara*D139 Δ(*ara*A-*leu*)7697 *gal*U *gal*K *rps*L *end*A1 *nup*G | Invitrogen |
| CS703-1 | Pentapeptide rich strain: CS109 ∆*mrcA* ∆*dacB* ∆*dacA* ∆*dacC* ∆*pbpG* ∆*ampC* ∆*ampH* | [9] |
| ***Acinetobacter baumannii*** | | |
| TCDC-AB0715 | Clinical isolate of *A.baumannii* identified from 16S rDNA sequencing | This study; Gift from Dr. R Bayston, University of Nottingham |
| ***Bdellovibrio bacteriovorus*** | | |
| HD100 | Type strain, genome sequenced | [10,11] |
| HD100 ∆*bd0816* | HD100 with a silent deletion of *bd0816* | This study |
| HD100 ∆*bd3459* | HD100 with a silent deletion of *bd3459* | This study |
| HD100 ∆*bd0816* ∆*bd3459* | HD100 ∆*bd3459* with a silent deletion of *bd0816* | This study |
| **Plasmids** | | |
| pK18*mobsacB* | Km^r^ suicide vector used for conjugation and recombination into *Bdellovibrio* genome | [12] |
| pZMR100 | λ defective vector, Km^r^. Used to confer Km^r^ on S17-1 used as prey | [7] |
| pUC19 | Amp^r^ cloning vector | [13] |
| pK18::∆*bd0816* | pK18*mobsacB* suicide plasmid containing 1kb of 5’- and 3’- flanking genomic DNA from around *bd0816* | This study |
| pK18::∆*bd3459* | pK18*mobsacB* suicide plasmid containing 1kb of 5’- and 3’- flanking genomic DNA from around *bd3459* | This study |
| pBADHisA | Amp^r^ plasmid with tightly regulated *araC* promoter and His-tag | Invitrogen |
| pBADKan::Bd3459 | Derivative of pBADHisA with His-tag removed, Km^r^ cassette inserted, and *bd3459* reading frame placed under control of *araC* promoter | This study |
| pBADKan::Bd3459(S70A) | Derivative of pBADHisA with His-tag removed, Km^r^ cassette inserted, and *bd3459* reading frame placed under control of *araC* promoter with a S70A site directed mutation | This study |
| pET41bct::Bd3459 | Km^r^ expression vector with GST tag removed and secreted form of Bd3459 inserted | This study |

**Table S2.** PCR primers used in this study.

| **Primer Name** | **Sequence 5’-3’** | **Description** |
| --- | --- | --- |
| Bd3244RT-F | CTTTAAGAACATCACGCAGC | F primer to amplify an 87 bp fragment of *dacB* (*bd3244*) from DNA originating from RNA isolated throughout the HD100 invasion cycle |
| Bd3244RT-R | GTACCGGTGAGTTGAATTCC | R primer to amplify an 87 bp fragment of *dacB* (*bd3244*) from DNA originating from RNA isolated throughout the HD100 invasion cycle |
| Bd0816RT-F | TCTGCACCTGACTCCAACAG | F primer to amplify a 120 bp fragment of *bd0816* from DNA originating from RNA isolated throughout the HD100 invasion cycle |
| Bd0816RT-R | ACCTTCATGCGGTTCAGTTC | R primer to amplify a 120 bp fragment of *bd0816* from DNA originating from RNA isolated throughout the HD100 invasion cycle |
| Bd3459RT-F | CCGCTTCTGCGAAAGTTTAC | F primer to amplify an 88 bp fragment of *bd3459* from DNA originating from RNA isolated throughout the HD100 invasion cycle |
| Bd3459RT-R | TCTTTGTTGTCATCGCCTTG | R primer to amplify an 88 bp fragment of *bd3459* from DNA originating from RNA isolated throughout the HD100 invasion cycle |
| Bd0816-F | ATCAGAATTCCCGGACTGTCGAAGTTCACC | F primer to amplify approximately 1 kb upstream flanking DNA from *bd0816* gene with *EcoRI* site added |
| Bd0816-R | TGATGTCGACGGCAAGTACCAGGCATGGTTTCC | R primer to amplify approximately 1 kb downstream flanking DNA from *bd0816* gene with *SalI* added |
| Bd0816-∆F | GACGTATGGCTTCGAAGAAGTAGGGATCTCTTTAGAAGGGG | Internal primer with homology to 5’ start of gene and 3’ end of gene |
| Bd0816-∆R | AAAGAGGATCCCTACTTCTTCGAAGCCATACGTCCTCCTATGC | Internal primer with homology to 5’ start of gene and 3’ end of gene |
| Bd3459-F | ATCAGAATTCCCATCTCGCCGATCTGAAAGGC | F primer to amplify approximately 1 kb upstream flanking DNA from *bd3459* gene with *EcoRI* site added |
| Bd3459-R | TGATGTCGACCCAAGAAGACAAGAGGGTGGTCCG | R primer to amplify approximately 1 kb downstream flanking DNA from *bd3459* gene with *SalI* site added |
| Bd3459-∆F | AAATCAAATACAAGAAATAGGGATCCGTGACGTATGAAAAAA | Internal primer with homology to 5’ start of gene and 3’ end of gene |
| Bd3459-∆R | GTCACGGATCCCTATTTCTTGTATTTCATGATTTGTACCACC | Internal primer with homology to 5’ start of gene and 3’ end of gene |
| F-3459forpBAD | GGCCATGGGGAAATACAGCCAGTGTCTCAAAGG | F primer to amplify *bd3459* gene from HD100 chromosome with *NcoI* site added |
| 3459STOP-HindIII | CGACAGAGAAGAAATAGAAGCTTGG | R primer to amplify *bd3459* from HD100 chromosome with *HindIII* site added |
| Bd3459-S70A-F | CCCTTTGGCTGCCATCTCCAAGG | F primer to make a S70A mutation in *bd3459* |
| Bd3459-S70A-R | CCTTGGAGATGGCAGCCAAAGGG | R primer to make a S70A mutation in *bd3459* |
| 3459CHispET41ctF | GTTTAACTTTAAGAAGGAGATATACATATGAAAGTTTACTTGAATTCCATGTGC | F primer to amplify secreted form of *bd3459* and provide homology to pET41 vector |
| 3459CHispET41ctR | GTGGTGGTGGTGGTGGTGCTCGAGTTTCTTCTCTGTCGTGATAGTGTTC | R primer to amplify secreted form of *bd3459* and provide homology to pET41 vector |

**References for Text S1 and Table S1-S2.**

1. Roschanski N, Klages S, Reinhardt R, Linscheid M, Strauch E (2011) Identification of genes essential for prey-independent growth of *Bdellovibrio bacteriovorus* HD100. J Bacteriol 193: 1745-1756.

2. Steyert SR, Pineiro SA (2007) Development of a novel genetic system To create markerless deletion mutants of *Bdellovibrio bacteriovorus*. Appl Environ Microbiol 73: 4717-4724.

3. Lambert C, Smith MCM, Sockett RE (2003) A novel assay to monitor predator–prey interactions for *Bdellovibrio bacteriovorus* 109 J reveals a role for methyl-accepting chemotaxis proteins in predation. Environ Microbiol 5: 127-132.

4. van den Ent F, Lowe J (2006) RF cloning: a restriction-free method for inserting target genes into plasmids. J Biochem Biophys Methods 67: 67-74.

5. Simon R, Priefer U, Puhler A (1983) A broad host range mobilization system for *in vivo* genetic engineering: transposon mutagenesis in Gram negative bacteria. Nat Biotech 1: 784-791.

6. Hanahan D (1983) Studies on transformation of *Escherichia coli* with plasmids. J Mol Biol 166: 557-580.

7. Rogers M, Ekaterinaki N, Nimmo E, Sherratt D (1986) Analysis of Tn7 transposition. Mol Gen Genet 205: 550-556.

8. Blattner FR, Plunkett G, Bloch CA, Perna NT, Burland V, et al. (1997) The complete genome sequence of *Escherichia coli* K-12. Science 277: 1453-1462.

9. Meberg BM, Sailer FC, Nelson DE, Young KD (2001) Reconstruction of *Escherichia coli* mrcA (PBP 1a) mutants lacking multiple combinations of penicillin binding proteins. J Bacteriol 183: 6148-6149.

10. Stolp H, Starr MP (1963) *Bdellovibrio bacteriovorus* gen. et sp. n., a predatory, ectoparasitic, and bacteriolytic microorganism. Antonie van Leeuwenhoek 29: 217-248.

11. Rendulic S, Jagtap P, Rosinus A, Eppinger M, Baar C, et al. (2004) A predator unmasked: life cycle of *Bdellovibrio bacteriovorus* from a genomic perspective. Science 303: 689-692.

12. Schäfer A, Tauch A, Jäger W, Kalinowski J, Thierbach G, et al. (1994) Small mobilizable multi-purpose cloning vectors derived from the *Escherichia coli* plasmids pK18 and pK19: selection of defined deletions in the chromosome of *Corynebacterium glutamicum*. Gene 145: 69-73.

13. Yanisch-Perron C, Vieira J, Messing J (1985) Improved M13 phage cloning vectors and host strains: nucleotide sequences of the M13mpl8 and pUC19 vectors. Gene 33: 103-119.
